# Obstetric and neonatal outcomes in pregnant women with idiopathic polyhydramnios: a systematic review and meta-analysis

**DOI:** 10.1038/s41598-024-54840-0

**Published:** 2024-03-04

**Authors:** Konstantinos S. Kechagias, Konstantinos Katsikas Triantafyllidis, Georgia Zouridaki, Makrina Savvidou

**Affiliations:** 1 Department of Metabolism, Digestion and Reproduction & Department of Surgery and Cancer, Faculty of Medicine, IRDB, Imperial College London, Hammersmith Campus, Du Cane Road, 3rd Floor, London, W12 0NN UK; 2 Department of Nutrition and Dietetics, Royal Marsden Hospital, London, UK; 3 Fetal Medicine Unit, Department of Obstetrics and Gynaecology, Chelsea and Westminster Hospital, London, UK

**Keywords:** Outcomes research, Reproductive signs and symptoms, Risk factors

## Abstract

Although the assessment of the amniotic fluid volume in pregnancy is part of the fetal wellbeing surveillance, the impact of idiopathic polyhydramnios (IP) on maternal and perinatal outcomes in unknown. The aim of this meta-analysis was to investigate the association of IP with different maternal and perinatal outcomes. We screened five electronic databases until December 2023 and performed data extraction and quality assessment using ROBINS-E in duplicates. Pooled risk ratios and 95% confidence intervals (95% CI) were calculated with a random effects model. 38 studies were included. Patients with IP were at increased risk of perinatal complications including preterm delivery (RR 1.96, 95% CI 1.35–2.86; I^2^ = 92%), placental abruption (RR 3.20, 95% CI 2.20–4.65; I^2^ = 2%), delivery via caesarean section (RR 1.60, 95% CI 1.39–1.84; I^2^ = 95%) and postpartum haemorrhage (RR 1.98, 95% CI 1.22–3.22; I^2^ = 84%). Similarly, IP was associated with increased risk of adverse perinatal outcomes including low APGAR score (RR 3.0, 95% CI 1.23–7.35; I^2^ = 95%), stillbirth (RR 4.75, 95% CI 2.54–8.86; I^2^ = 9%) and perinatal mortality (RR 4.75, 95% CI 2.67–8.48; I^2^ = 37%). This meta-analysis suggests that pregnant women with IP may be at increased risk of perinatal complications and adverse neonatal outcomes. However, data remains inconclusive considering the low quality and high heterogeneity of included studies.

*PROSPERO registration number*: CRD42022359944.

## Introduction

Assessment of the amniotic fluid volume (AFV) in pregnancy is part of the surveillance of fetal wellbeing and polyhydramnios constitutes the abnormal increase in amniotic fluid volume^1^. It is defined as the presence of a single deepest vertical pocket (DVP) of  ≥ 8 cm or an amniotic fluid index (AFI) of  ≥ 25 cm^2^. Although, measurement of AFI, compared to DVP, has been shown to increase the diagnosis of reduced amniotic fluid and rates of medical intervention, without improving perinatal outcomes, both definitions continue to be used interchangeably. Polyhydramnios is classified as mild, moderate or severe (DVP = 8–11, 12–15,  ≥ 16 and/or AFI = 25–29.9, 30–34.9,  ≥ 35 cm, respectively) depending on the amount of amniotic fluid present^1^. The condition affects almost 1–2% of singleton pregnancies although data regarding prevalence are inconsistent mainly due to the diagnostic criteria used, the population studied and the frequency of ultrasound scans^3^.

Although polyhydramnios can be associated with chromosomal abnormalities, genetic syndromes, fetal structural malformations, anaemia, infections, placental abnormalities and maternal diabetes, in the majority of the cases (70%) no reason is identified and the polyhydramnios is classified as idiopathic^4^. Idiopathic polyhydramnios is usually diagnosed in the third trimester of pregnancy and its impact on maternal and perinatal outcomes is a matter of debate^5^. Consequently, the antenatal management of pregnant women presenting with idiopathic polyhydramnios constitutes a challenge for obstetricians and feto-maternal medicine specialists. Several studies suggest that antenatal fetal surveillance is not required for cases of mild polyhydramnios and that spontaneous delivery should be planned^1^. On the other hand, other studies recommend increased antenatal surveillance and delivery at 39 weeks, due to the theoretical increased risk of adverse pregnancy outcomes associated with idiopathic polyhydramnios^4,6^.

The aim of this systematic review and meta-analysis was to systematically screen the existing literature and explore the impact of idiopathic polyhydramnios on different maternal and perinatal outcomes.

## Methods

This systematic review follows the PRISMA reporting guidelines^7^. The protocol was registered in PROSPERO (CRD42022359944).

### Literature search

We searched PubMed/MEDLINE, Scopus, Cochrane central, Web of Science and ClinicalTrials.gov from inception until December 2023. Two authors (KSK and KKT) developed the search strategy and screened the articles independently. The following terms were used: (Idiopathic OR isolated) AND (polyhydramnios OR hydramnios). No language or geographic restrictions were set. Grey literature and reference lists of relevant reviews and articles selected for inclusion were manually searched for relevant publications possibly missed during the initial screening. Discrepancies in the search process were discussed and resolved by MS.

### Eligibility criteria

The systematic review included all studies reporting on perinatal outcomes in women with idiopathic polyhydramnios. In the meta-analysis, we only included studies that also reported results from a cohort of patient with normal amniotic fluid volume. Studies were included irrespective of study design.

Although published dissertations were excluded from the literature search, since they usually contain preliminary or incomplete data, abstracts submitted in conferences were considered eligible. Google scholar screening, customised Google searches and consultation with experts were also used to identify articles in the grey literature.

### Data extraction and risk of bias

Data were independently extracted by KSK and GZ in pre-specified forms. Discrepancies were discussed and resolved by KKT and MS. Risk of bias (RoB) was assessed by two authors independently (KSK and KKT) using ROBINS-E tool (Risk Of Bias In Non-randomised Studies–of Exposures) for observational studies.

The GRADE (Grading of Recommendations, Assessment, Development and Evaluations) approach was used to assess the quality of evidence of the main outcomes. The assessment was based on five parameters: risk of bias, inconsistency (known also as heterogeneity) between studies, indirectness, imprecision (risk of random errors), and publication bias. The evidence for each item was rated as high, moderate, low, or very low.

### Definitions of outcome

Our study examined the following outcomes: (1) preterm birth (2) preterm premature rupture of membranes (PPROM) (3) pregnancy induced hypertension/pre-eclampsia (PIH/PE) (4) malpresentation (5) transverse lie (6) breech presentation (7) labour induction (8) epidural analgesia (9) cephalopelvic disproportion (10) cord prolapse (11) placental abruption (12) shoulder dystocia (13) caesarean section (14) post-partum haemorrhage (PPH) (15) low birth weight (16) macrosomia (17) 1 min appearance, pulse, grimace, activity, and respiration (APGAR) score (18) 5 min APGAR score (19) neonatal intensive care unit (NICU) admission (20) stillbirth and (21) perinatal mortality. The aforementioned outcomes were divided and presented in three groups namely antenatal complications, intrapartum/postpartum complications and neonatal outcomes. The definition of outcomes in the current meta-analysis was based on the definitions provided by the individual studies (supplementary material).

### Statistical analysis

We combined study effect sizes using risk ratios (RR) along with corresponding 95% confidence intervals (95% CIs) under the random-effects meta-analysis model and the inverse variance method. Statistical heterogeneity was assessed by using the x^2^ test (*p* < 0.10 to indicate statistically significant heterogeneity) and I^2^ (to quantify the degree of heterogeneity). I^2^ from 30 to 49% was defined as moderate heterogeneity and 50% or more was defined as high heterogeneity for the data^8^. The Der Simonian and Laird estimator was used to estimate the between-study variance. Visual inspection of the funnel plot was used to assess for publication bias when more than ten studies were available for each outcome.

To explore the possible sources of heterogeneity, we performed predefined sensitivity analyses for every outcome restricting to: studies with moderate or low risk of bias. Given that our meta-analysis examined rare events and due to possible publication bias, we also performed fixed-effects (FE) meta-analysis using the Mantel–Haenszel method; this approach provides more reliable estimates of the summary effect, at the cost of ignoring heterogeneity.

We further conducted a series of subgroup analyses to explore sources of heterogeneity and differences in summary estimates according to: continent (Europe vs Americas vs Asia), mean maternal age (> 30 years vs  ≤ 30 years), study design (retrospective vs prospective), definition of idiopathic polyhydramnios (DVP vs AFI vs mixed criteria), year of study (> 2010 vs  ≤ 2010), mean birth weight (< 3 kg vs 3–3.5 kg vs  > 3.5 kg) and gestational week at diagnosis (> 26 weeks vs  ≤ 26 weeks).

The meta-analysis was performed using RevMan (Review Manager) Web in the online platform provided for Cochrane intervention reviews (RevMan, Copenhagen: The Nordic Cochrane Centre, the Cochrane Collaboration, 2008).

## Results

### Characteristics of the included studies

**Figure 1:**
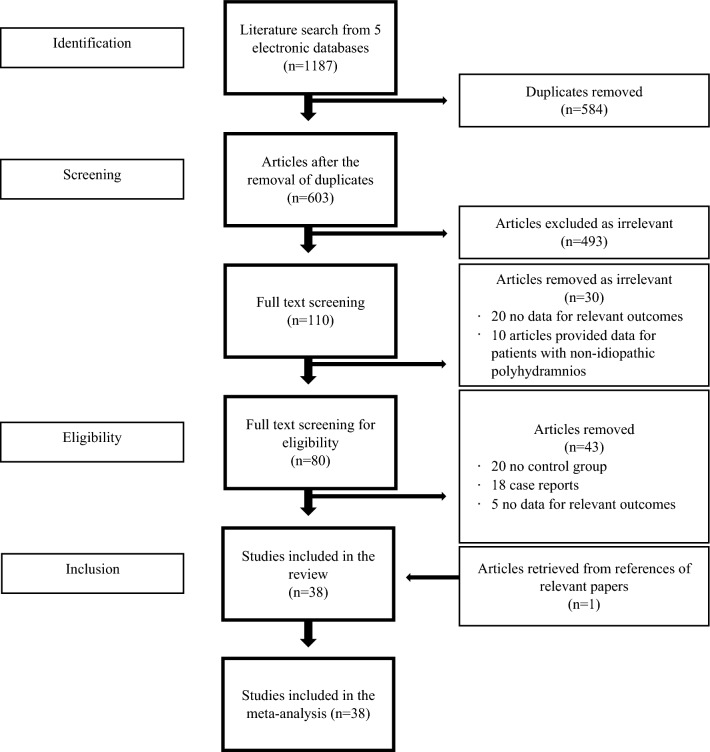
PRISMA flowchart.

The initial literature search yielded 1187 articles. After the exclusion of duplicates and irrelevant titles, 110 full texts were screened, and 38 studies were found eligible for the systematic review (Fig. 1)^3,9–45^. All 38 studies were observational; 29 were cohort studies, three were case–control studies. The design of six studies were not stated. The majority of the studies (26/38) explored idiopathic polyhydramnios as part of a number of risk factors associated with pregnancy and neonatal outcomes. Eleven studies were conducted in Europe, nine studies in Americas and 18 in Asia (supplementary material).

### Risk of bias

The majority of the included studies (36/38) were classified as of low or moderate risk of bias with adequate overall design (ROBINS-E tool). However, some concerns were identified related to the exposure window and selection of participants (26/38) as well as to the selection of reported results (2/38). Additionally, two studies (2/38) were characterised as of high risk of bias due to the potential presence of uncontrolled confounding factors (supplementary material).

### Antenatal complications

**Figure 2:**
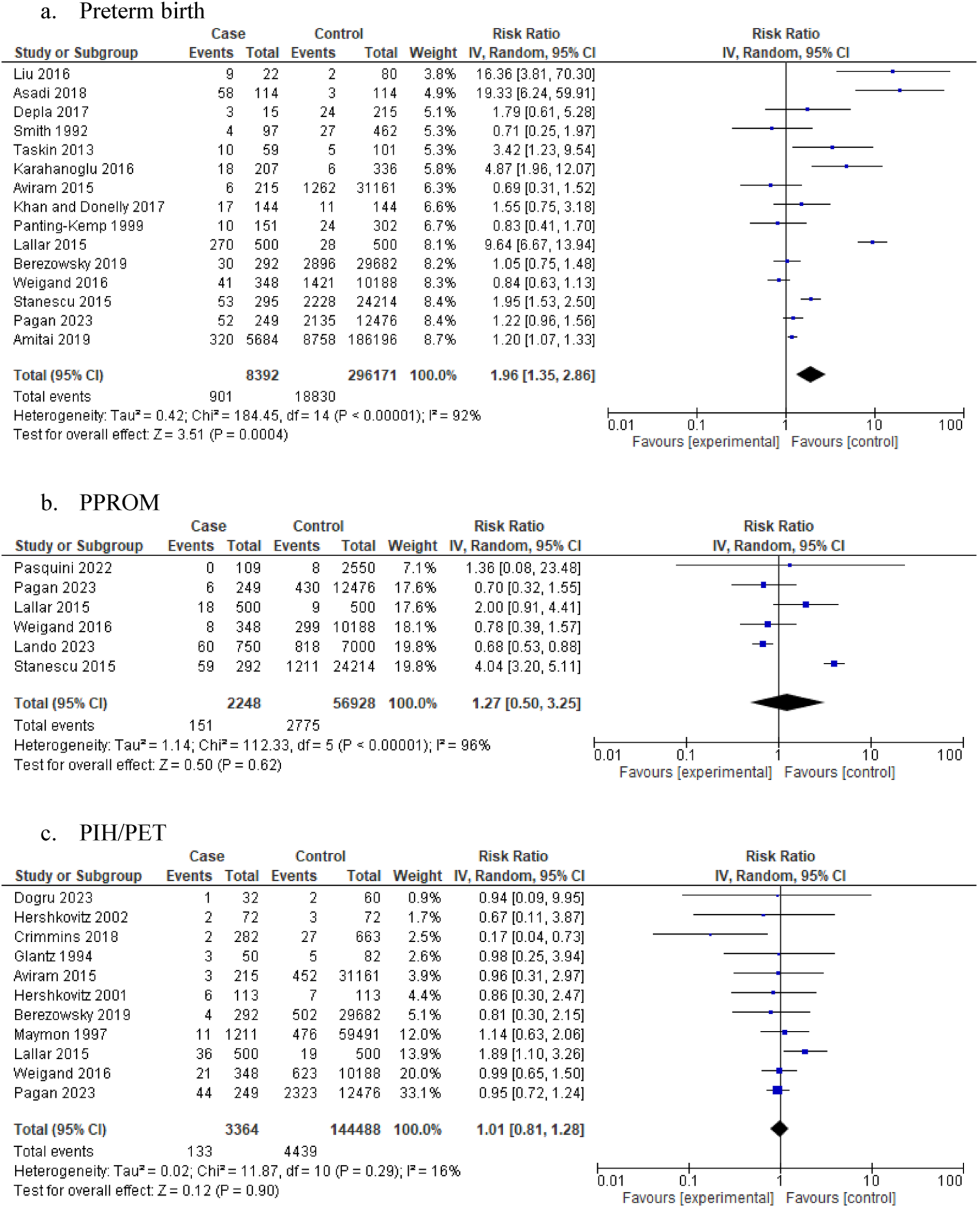

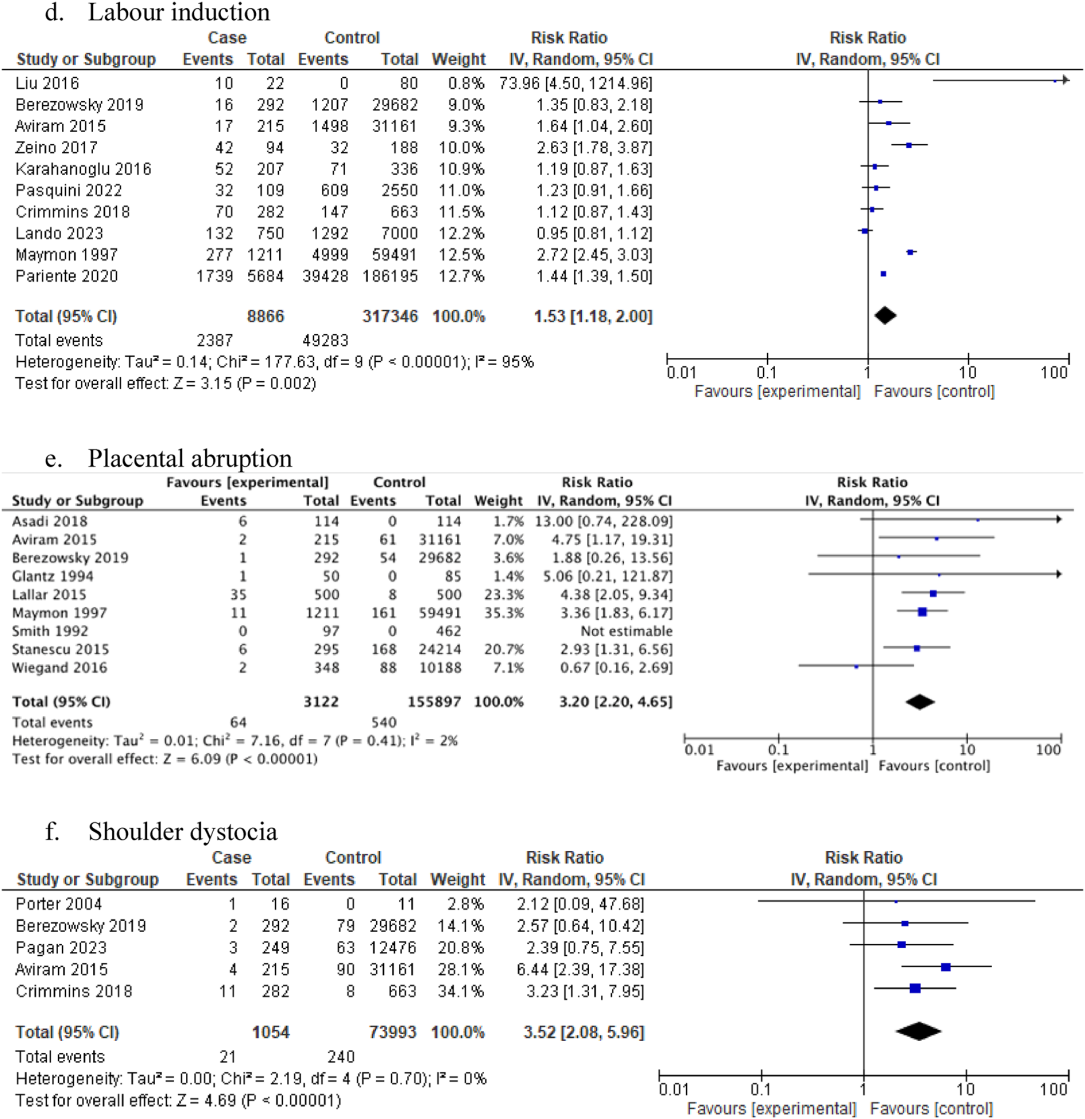

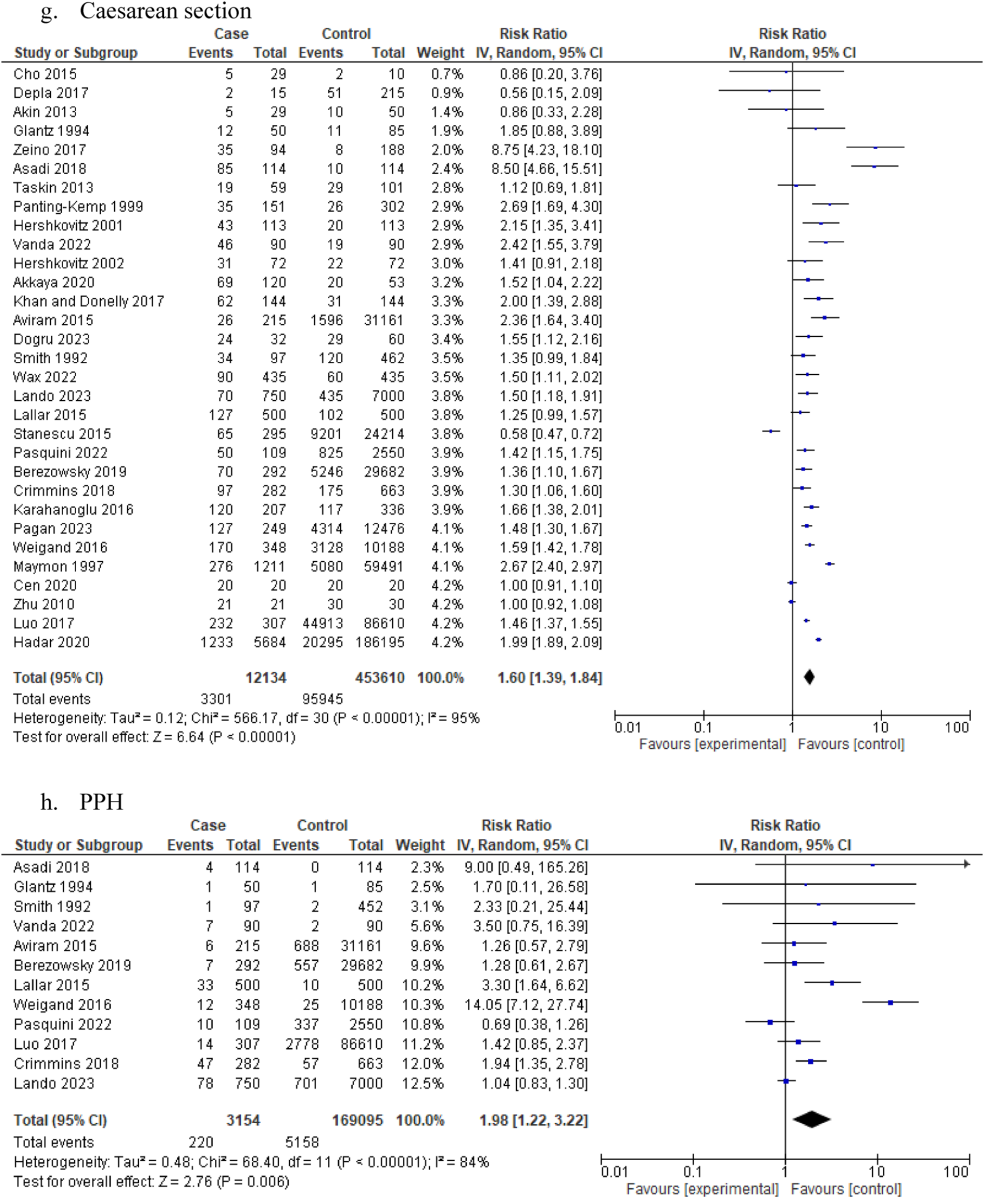

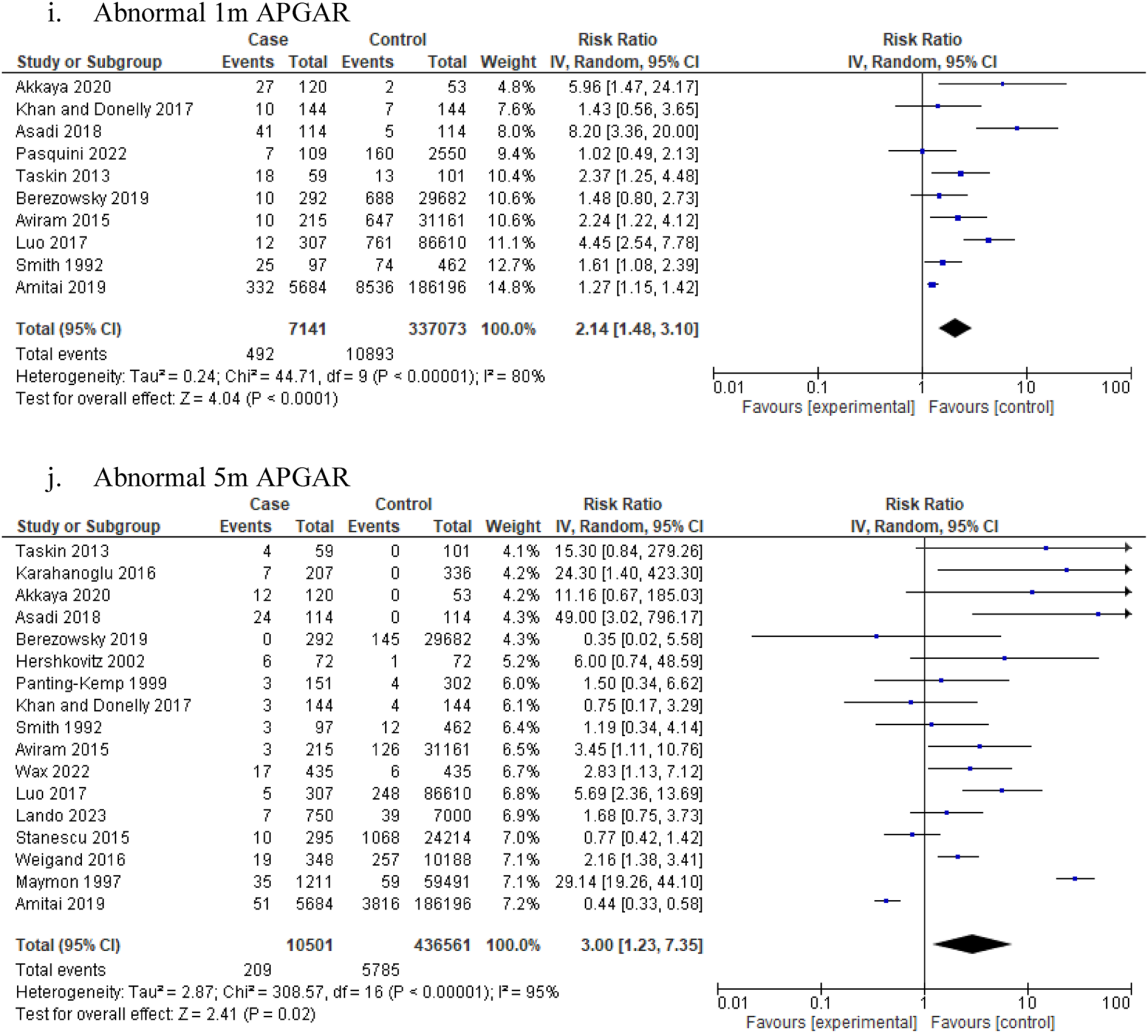

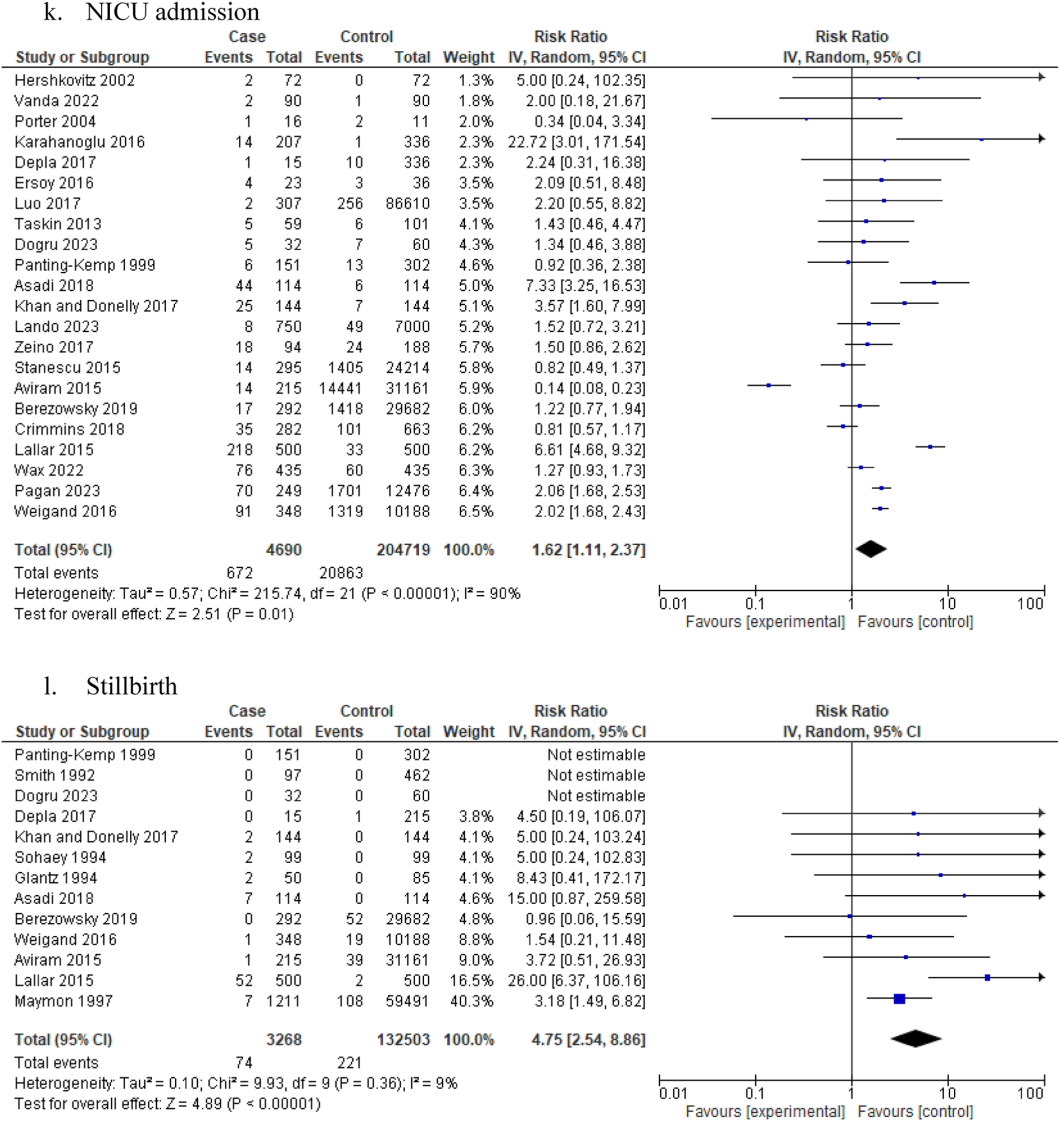

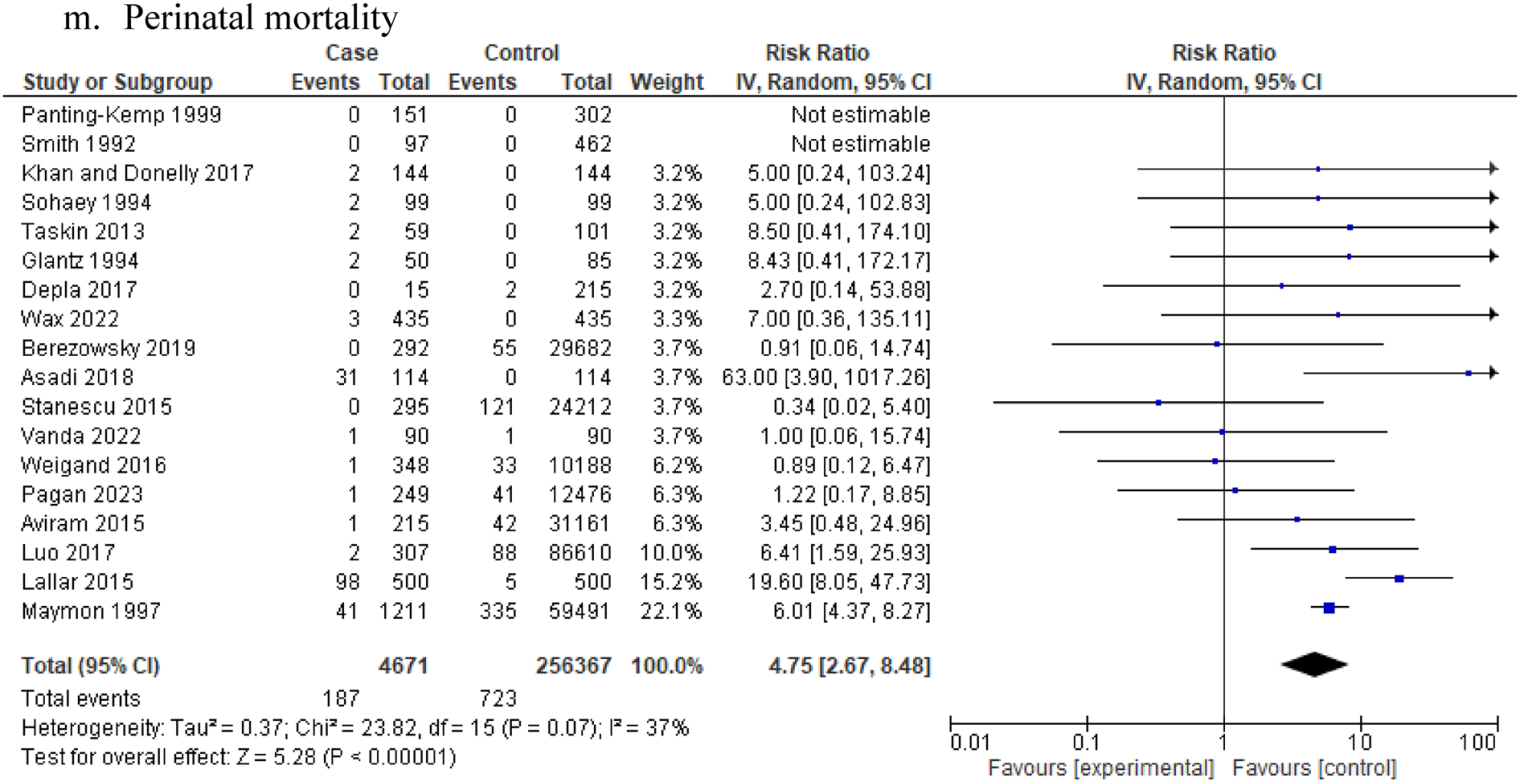
Association of idiopathic polyhydramnios with the risk of (**a**) Preterm birth (**b**) PPROM (**c**) PIH/PET (**d**) Labour induction (**e**) Placental abruption (**f**) Shoulder dystocia (**g**) Caesarean section (**h**) PPH (**i**) Abnormal 1 m APGAR (**j**) Abnormal 5 m APGAR (**k**) NICU admission (**l**) Stillbirth (**m**) Perinatal mortality. *1 m*: 1 min/*5 m*: 5 min; *95% CI* 95% confidence interval; *APGAR* appearance, pulse, grimace, activity, and respiration score; *IP* idiopathic polyhydramnios; *NICU* neonatal intensive care unit; *PIH/PET* pregnancy induced hypertension/pre-eclamptic toxaemia; *PPH* post-partum haemorrhage; *PPROM* preterm premature rapture of membranes; *RR* risk ratio; *IV* inverse variance.

Our analysis showed that women with idiopathic polyhydramnios were at increased risk of delivering preterm compared to women with normal AFV (15 studies: 304,563 participants; RR 1.96, 95% CI 1.35–2.86; I^2^ = 92%, τ^2^ = 0.42) (Table 1, Fig. 2a). Confidence in this meta-analysis was low according to GRADE due to the presence of inconsistency as a result of high heterogeneity and non-overlapping confidence intervals.

**Table 1:** Summary of findings.

Outcome	No of studies	Total number of participants	Idiopathic polyhydramnios group events per total n/N (%)	Non-idiopathic polyhydramnios group events per total n/N (%)	RR (95% CI) [random effect model-inverse variance method]	I^2^	τ^2^
Antenatal complications (n = 3)							
Preterm birth	15	304,563	901/8392 (10.7%)	18,830/296,171 (6.3%)	1.96 [1.35, 2.86]	92%	0.42
PPROM	6	59,176	151/2248 (6.7%)	2775/56,928 (4.8%)	1.27 [0.50, 3.25]	96%	1.14
PIH/PE	11	147,852	133/3364 (3.9%)	4439/144,488 (3%)	1.01 [0.81, 1.28]	16%	0.02
Intrapartum/peripartum complications (n = 11)							
Malpresentation	15	212,008	294/4258 (6.9%)	7793/207,750 (3.7%)	1.82 [1.42, 2.35]	54%	0.10
Transverse lie	2	1160	33/559 (5.9%)	13/601 (2.2%)	2.64 [1.29, 5.42]	6%	0.03
Breech	2	30,974	27/792 (3.4%)	1083/30,182 (3.6%)	1.34 [0.52, 3.46]	70%	0.33
Labour induction	10	326,212	2387/8866 (28.9%)	49,283/317,346 (17.6%)	1.53 [1.18, 2.00]	95%	0.14
Epidural analgesia	3	69,100	880/1257 (70.0%)	43,410/67,843 (63.9%)	1.08 [0.98, 1.18]	81%	0.01
Cephalopelvic disproportion	5	2235	44/946 (4.7%)	26/1289 (2.0%)	2.43 [1.02, 5.84]	53%	0.48
Cord prolapse	11	151,925	43/3797 (1.1%)	343/148,128 (0.2%)	6.5 [4.68, 9.04]	0%	0.00
Placental abruption	9	159,019	64/3122 (2.0%)	540/155,897 (0.3%)	3.20 [2.20, 4.65]	2%	0.01
Shoulder dystocia	5	75,047	21/1054 (2%)	240/73,993 (0.3%)	3.52 [2.08, 5.96]	0%	0
Caesarean section	31	465,744	3301/12,134 (27.2%)	95,945/453,610 (21.1%)	1.60 [1.39, 1.84]	95%	0.12
PPH	12	172,249	220/3154 (6.9%)	5158/169,095 (3.0%)	1.98 [1.22, 3.22]	84%	0.48
Neonatal outcomes (n = 7)							
Low birth weight	7	304,434	267/6754 (4.0%)	12,225/297,680 (4.1%)	1.21 [0.74, 1.98]	82%	0.31
Macrosomia	20	546,459	2001/15,586 (12.8%)	27,506/530,873 (5.1%)	2.68 [2.43, 2.95]	48%	0.01
1 min APGAR	10	344,214	492/7141 (6.9%)	10,893/337,073 (3.2%)	2.14 [1.48, 3.10]	80%	0.24
5 min APGAR	17	447,062	209/10,501 (2.0%)	5785/436,561 (1.3%)	3.00 [1.23, 7.35]	95%	2.87
NICU admission	22	209,409	672/4690 (14.3%)	20,863/204,719 (10.1%)	1.62 [1.11, 2.37]	90%	0.57
Stillbirth	13	135,771	74/3268 (2.2%)	221/132,503 (0.1%)	4.75 [2.54, 8.86]	9%	0.10
Perinatal mortality	18	261,038	187/4671 (3.4%)	723/256,367 (0.3%)	4.75 [2.67, 8.48]	37%	0.37

*1 m*: 1 min/*5 m*: 5 min; *APGAR* appearance, pulse, grimace, activity, and respiration score; *IP* idiopathic polyhydramnios; *NICU* neonatal intensive care unit; *PIH/PE* pregnancy induced hypertension/pre-eclampsia; *PPH* post-partum haemorrhage; *PPROM* preterm premature rapture of membranes.

In women with idiopathic polyhydramnios, the risk of PPROM (6 studies: 59,176 participants; RR 1.27, 95% CI 0.50–3.25; I^2^ = 96%, τ^2^ = 1.14) (Table 1, Fig. 2b) and PIH/PE (11 studies: 147,852participants; RR 1.01, 95% CI 0.81–1.28; I^2^ = 16%, τ^2^ = 0.02) (Table 1, Fig. 2c) was similar compared to women with normal AFV.

### Intrapartum/postpartum complications

Women with idiopathic polyhydramnios were at increased risk of labour induction (10 studies: 326,212 participants; RR 1.53, 95% CI 1.18–2.00; I^2^ = 95%, τ^2^ = 0.14) (Table 1, Fig. 2d) and placental abruption (9 studies: 159,019 participants; RR 3.20, 95% CI 2.20–4.65; I^2^ = 2%, τ^2^ = 0.01) (Table 1, Fig. 2e) compared to women with normal AFV. Low heterogeneity and low certainty were observed.

Similarly, women with idiopathic polyhydramnios were at increased risk of shoulder dystocia (5 studies: 75,047participants; RR 3.52, 95% CI 2.08–5.96; I^2^ = 0%, τ^2^ = 0) (Table 1, Fig. 2f) and delivering via a caesarean section compared to women with normal AFV (31 studies: 465,744 participants; RR 1.60, 95% CI 1.39–1.84; I^2^ = 95%, τ^2^ = 0.12) (Table 1, Fig. 2g). High heterogeneity and very low certainty based on GRADE were observed in both outcomes. Regarding the risk of delivering via a caesarean section the summary effect estimate remained similar in the subgroup analyses according to study design and mean birthweight (supplementary material). Women with idiopathic polyhydramnios were also at increased risk of PPH compared to controls (12 studies: 172,249 participants; RR 1.98, 95% CI 1.22–3.22; I^2^ = 84%, τ^2^ = 0.48) (Table 1, Fig. 2h) although high heterogeneity was observed. A similar summary effect estimate on the risk of PPH was also noticed in the subgroup analyses according to mean birthweight (supplementary material).

### Neonatal outcomes

The presence of idiopathic polyhydramnios was associated with increased risk of APGAR score < 7 at 1 min (10 studies: 344,214 participants; RR 2.14, 95% CI 1.48–3.10; I^2^ = 80%, τ^2^ = 0.24) (Table 1, Fig. 2i) and 5 min (17 studies: 447,062 participants; RR 3.00, 95% CI 1.23–7.35; I^2^ = 95%, τ^2^ = 2.87) (Table 1, Fig. 2j). Confidence in these meta-analyses ranged from very low to low according to GRADE. The risk of NICU admission was also increased among patients with idiopathic polyhydramnios (22 studies: 209,409 participants; RR 1.62, 95% CI 1.11–2.37; I^2^ = 90%, τ^2^ = 0.57) (Table 1, Fig. 2k).

Furthermore, the risk of stillbirth was increased in women with idiopathic polyhydramnios compared to pregnant women with normal AFV (13 studies: 135,771 participants; RR 4.75, 95% CI 2.54–8.86; I^2^ = 9%, τ^2^ = 0.10) (Table 1, Fig. 2l). Low to moderate heterogeneity and low certainty based on GRADE were observed. In pregnancies with idiopathic polyhydramnios, the risk of perinatal mortality was also increased compared to pregnancies with normal AFV (18 studies: 261,038 participants; RR 4.75, 95% CI 2.67–8.48; I^2^ = 37%, τ^2^ = 0.37) (Table 1, Fig. 2m) while low heterogeneity was observed.

## Discussion

### Overview

The findings of this meta-analysis suggest that pregnant women with idiopathic polyhydramnios may be at increased risk of perinatal complications including preterm delivery, labour induction, placental abruption, shoulder dystocia, delivery via caesarean section, and postpartum haemorrhage. Additionally, our analysis showed that idiopathic polyhydramnios may potentially increase the risk of adverse perinatal outcomes including low 1-min and 5-min APGAR scores, stillbirth and perinatal mortality. These results should be interpreted with caution due to the high heterogeneity observed in the majority of studied outcomes affecting the overall quality of evidence.

### Findings in the context of the literature

Although pregnancies complicated by idiopathic polyhydramnios are characterised as high risk^42^, there is still no agreement in regards to specific complications associated with the condition. As a result, reports in the literature suggest that antenatal fetal surveillance is not required in cases of idiopathic polyhydramnios^1^. The included studies have used either AFI or DVP. Nevertheless, the fact that the available studies did not provide data according to polyhydramnios severity and their results are inconsistent demonstrates that the condition deserves further study as a potential risk factor for adverse perinatal outcomes.

One previous meta-analysis attempted to synthetise the evidence to date and suggested induction of labour at 39 weeks in pregnancies complicated by idiopathic polyhydramnios^46^. Our findings on the summary estimates are in agreement with this study, which, however, only included a fraction of the published literature, failed to include grey literature and unpublished data in the search and implemented exclusion criteria that affected the final number of studies with potentially relevant data. Additionally the presence of significant inconsistency was not explored and was not associated with the overall quality of the data. GRADE assessment was not performed, whilst the risk of bias tool used (Newcastle–Ottawa Scale) is not recommended by the Cochrane Library^46^.

From a pathophysiological point of view, increased AFV can cause uterine overdistention which can potentially explain the increased risk of preterm labour, placental abruption and PPH as the uterus becomes less responsive to oxytocin, leading to uterine atony^26^. Uterine overdistention and increased intrauterine pressure could also lead to a degree of ‘placental insufficiency’ and this could explain the altered feto-placental Doppler velocimetry seen in patients diagnosed with idiopathic polyhydramnios^34,47^. This putative mechanism could represent a possible link between idiopathic polyhydramnios and adverse neonatal outcomes including low APGAR scores and perinatal mortality.

### Strengths and limitations

To our knowledge, this is the most comprehensive systematic review and meta-analysis to report on the effect of idiopathic polyhydramnios on perinatal outcomes with thorough methodological assessment of risk of bias, heterogeneity, and appropriate data synthesis.

We used a reliable risk of bias tool (ROBINS-E) to explore the quality of the included studies. We further explored the possibility of publication bias in our meta-analysis using the assessment of funnel plot symmetry. We performed a thorough assessment of the grey literature and we also assessed our findings using both fixed and random effects meta-analyses, as well as using a series of subgroup and sensitivity analyses that controlled for risk of bias, study design, birth weight, maternal age, year of publication, timing of diagnosis etc.

However, the findings should be interpreted with caution. The majority of the published literature comes from observational studies at risk of bias. These observational studies were of moderate quality based on the ROBINS-E tool and only a small fraction of them provided adjusted data. The moderate quality of the included articles should be attributed, at least partially, to the presence of confounders, the differences between intervention and control groups, especially regarding birthweight, and the suboptimal selection of participants. Specifically, the mean age of participants was not provided in most of the studies and age differences may impact on the outcomes that we investigated. Moreover, the high diversity amongst studies with regards to definition of exposure, definition of outcomes and length of follow-up could also influence the accuracy of the summary effect estimates. Additionally, heterogeneity related to intrapartum care and management should be also mentioned as a potential source of indication bias. An example includes epidural analgesia which can be affected by patients’ preferences. Regarding preterm labour, the differentiation between spontaneous and induced was not feasible due to lack of data from the included studies. Lastly, data regarding the mean gestational age of delivery was not provided by the included studies and as a result we were not able to perform additional analyses based on this confounder.

The observed heterogeneity in several meta-analyses was high, as indicated by the I^2^ estimates and the wide non-overlapping confidence intervals, highlighting the uncertainty around the observed effect estimates. While specific subgroup and sensitivity analyses showed consistent results and were able to reduce this heterogeneity to some extent, the number of included studies in these subgroups was low. Additionally, the difference in effect estimates between the random and fixed effect models also indicates the potential presence of publication bias. The assessment of publication bias was restricted by the limited number of studies for some comparisons further highlighting the need for prospectively designed studies with long follow up periods. Finally, the quality of evidence and the certainty related to our results ranged from very low to low as assessed by GRADE due to the observational nature of the included studies and the presence of inconsistency.

## Conclusions

The findings of this meta-analysis suggest that pregnant women with idiopathic polyhydramnios may be at increased risk of perinatal complications and adverse neonatal outcomes. However, the quality of evidence for the majority of the studied outcomes ranged from very low to low; as such, the data remain inconclusive. Large-scale high-quality prospectively designed studies are required to investigate more robustly the effect of idiopathic polyhydramnios on pregnancy outcomes and optimal time of delivery. Until further data is available, antenatal fetal surveillance could be considered in pregnancies complicated by this condition.

### Supplementary Information

Supplementary Tables.

## Data Availability

All data relevant to the study are included in the article or uploaded as supplementary information.

## References

[1] Dashe JS (2018). SMFM consult series# 46: Evaluation and management of polyhydramnios. Am. J. Obstet. Gynecol..

[2] Pri-Paz S (2012). Maximal amniotic fluid index as a prognostic factor in pregnancies complicated by polyhydramnios. Ultrasound Obstet. Gynecol..

[3] Panting-Kemp A (1999). Idiopathic polyhydramnios and perinatal outcome. Am. J. Obstet. Gynecol..

[4] Magann EF (2007). A review of idiopathic hydramnios and pregnancy outcomes. Obstet. Gynecol. Surv..

[5] Sagi-Dain L, Sagi S (2015). Chromosomal aberrations in idiopathic polyhydramnios: A systematic review and meta-analysis. Eur. J. Med. Genet..

[6] Sandlin AT, Chauhan SP, Magann EF (2013). Clinical relevance of sonographically estimated amniotic fluid volume: Polyhydramnios. J. Ultrasound Med..

[7] Page, M. et al. The PRISMA 2020 statement: An updated guideline for reporting systematic reviews. *MetaArXiv.* (2020). 10.1186/s13643-021-01626-4 PMC8008539 33781348

[8] Cumpston M (2019). Updated guidance for trusted systematic reviews: A new edition of the Cochrane handbook for systematic reviews of interventions. Cochrane Database Syst. Rev..

[9] Akkaya H, Büke B, Destegül E (2020). The effect of increased amnion volume severity on fetal Doppler indices and perinatal outcomes in idiopathic polyhydramnios. J. Matern. Fetal Neonatal Med..

[10] Cen J (2020). Comparative proteome analysis of amniotic fluids and placentas from patients with idiopathic polyhydramnios. Placenta.

[11] Hadar O (2020). Prenatal exposure to isolated amniotic fluid disorders and the risk for long-term cardiovascular morbidity in the offspring. Gynecol. Endocrinol..

[12] Pariente G (2020). Prenatal exposure to isolated amniotic fluid disorders and the risk for long-term endocrine morbidity of the offspring. Arch. Gynecol. Obstet..

[13] Amitai A (2019). The association between pregnancies complicated with isolated polyhydramnios or oligohydramnios and offspring long-term gastrointestinal morbidity. Arch. Gynecol. Obstet..

[14] Berezowsky A (2019). Transient isolated polyhydramnios and perinatal outcomes. Ultraschall in der Medizin-European Journal of Ultrasound.

[15] Asadi N (2018). Perinatal outcome in pregnancy with polyhydramnios in comparison with normal pregnancy in department of obstetrics at Shiraz University of Medical Sciences. J. Matern.-Fetal Neonatal Med..

[16] Crimmins S (2018). Polyhydramnios or excessive fetal growth are markers for abnormal perinatal outcome in euglycemic pregnancies. Am. J. Perinatol..

[17] Depla AL (2017). Polyhydramnios in isolated oral cleft pregnancies: Incidence and outcome in a retrospective study. Prenat. Diagn..

[18] Karahanoglu E (2017). The effect of the amniotic fluid index on the accuracy of ultrasonographic-estimated fetal weight. Ultrasound Q..

[19] Khan S, Donnelly J (2017). Outcome of pregnancy in women diagnosed with idiopathic polyhydramnios. Aust. N. Z. J. Obstet. Gynaecol..

[20] Luo Q-Q (2017). Idiopathic polyhydramnios at term and pregnancy outcomes: A multicenter observational study. J. Matern.-Fetal Neonatal Med..

[21] Zeino S (2017). Delivery outcomes of term pregnancy complicated by idiopathic polyhydramnios. J. Gynecol. Obstet. Hum. Reprod..

[22] Ersoy AO (2016). The association between N-terminal pro-brain natriuretic peptide levels in the umbilical vein and amniotic fluid volume abnormalities. Rev. Bras. Ginecol. Obstet..

[23] Karahanoglu E (2016). Intrapartum, postpartum characteristics and early neonatal outcomes of idiopathic polyhydramnios. J. Obstet. Gynaecol..

[24] Liu L-L, Pang L-H, Deng B-Y (2016). Prenatal diagnosis and pregnancy outcome analysis of polyhydramnios. Fetal Pediatr. Pathol..

[25] Wiegand SL (2016). Idiopathic polyhydramnios: Severity and perinatal morbidity. Am. J. Perinatol..

[26] Aviram A (2015). Association of isolated polyhydramnios at or beyond 34 weeks of gestation and pregnancy outcome. Obstet. Gynecol..

[27] Cho GJ (2015). Decreased umbilical orexin-a level is associated with idiopathic polyhydramnios. Acta Obstet. Gynecol. Scand..

[28] Lallar M, ul Haq A, Nandal R (2015). Perinatal outcome in idiopathic polyhydramnios. J. Obstet. Gynecol. India.

[29] Stanescu A (2015). Idiopathic polyhydramnios and fetal gender. Arch. Gynecol. Obstet..

[30] Akin I (2013). Applicability of fetal renal artery Doppler values in determining pregnancy outcome and type of delivery in idiopathic oligohydramnios and polyhydramnios pregnancies. Ginekologia polska.

[31] Taskin S (2013). Perinatal outcomes of idiopathic polyhydramnios. Interv. Med. Appl. Sci..

[32] Zhu X (2010). The expression of aquaporin 8 and aquaporin 9 in fetal membranes and placenta in term pregnancies complicated by idiopathic polyhydramnios. Early Hum. Dev..

[33] Porter H, Lookinland S, Belfort MA (2004). Evaluation of a new real-time blood continuous glucose monitoring system in pregnant women without gestational diabetes. A pilot study. J. Perinat. Neonatal. Nurs..

[34] Hershkovitz R (2002). Uterine artery Doppler velocimetry in patients with idiopathic hydramnios. Fetal Diagn. Ther..

[35] Hershkovitz R (2001). Evidence for abnormal middle cerebral artery values in patients with idiopathic hydramnios. J. Matern. Fetal Med..

[36] Maymon E (1998). Isolated hydramnios at term gestation and the occurrence of peripartum complications. Eur. J. Obstet. Gynecol. Reprod. Biol..

[37] Glantz JC, Abramowicz JS, Sherer DM (1994). Significance of idiopathic midtrimester polyhydramnios. Am. J. Perinatol..

[38] Sohaey R (1994). Idiopathic polyhydramnios: Association with fetal macrosomia. Radiology.

[39] Smith CV (1992). Relation of mild idiopathic polyhydramnios to perinatal outcome. Obstet. Gynecol..

[40] Wax JR (2022). Transient idiopathic polyhydramnios: Maternal and perinatal outcomes. J. Ultrasound Med..

[41] Vanda R (2022). Comparing pregnancy, childbirth, and neonatal outcomes in women with idiopathic polyhydramnios: A prospective cohort study. BMC Pregnancy Childbirth.

[42] Pasquini L (2022). Obstetric and neonatal outcomes in mild idiopathic polyhydramnios. Children.

[43] Pagan M (2023). Is mild idiopathic polyhydramnios associated with an increased risk for an intrauterine fetal demise? A retrospective cohort study. Int. J. Women's Health.

[44] Bas Lando M (2023). Term idiopathic polyhydramnios, and labor complications. J. Clin. Med..

[45] Doğru Ş, Akkuş F (2023). Fetal epicardial fat thickness in non-severe idiopathic polyhydramnios: Its impact on fetal cardiac function and perinatal outcomes. J. Clin. Ultrasound.

[46] Pagan M (2023). Idiopathic polyhydramnios and pregnancy outcome: Systematic review and meta-analysis. Ultrasound Obstet. Gynecol..

[47] Fisk NM, Vaughan J, Talbert D (1994). Impaired fetal blood gas status in polyhydramnios and its relation to raised amniotic pressure. Fetal Diagn. Ther..

