# Supplementary material for: Obstetric and neonatal outcomes in pregnant women with idiopathic polyhydramnios: a systematic review and meta-analysis
Source: Sci Rep. 2024 Mar 4;14:5296. doi: 10.1038/s41598-024-54840-0 (PMC10912321; doi:10.1038/s41598-024-54840-0)
Supplement: Supplementary file 1 — Supplementary Tables. [file 41598_2024_54840_MOESM1_ESM.docx]

**SUPPLEMENTARY TABLES**

**Table S1.** Characteristics of the included studies.

| **Author**  **Year**  **Country** | **Study design** | **Age (IP cases)** | **Age (controls)** | **Number of IP cases** | **Number of controls** | **Definition of IP** | **Gestation at diagnosis** | **Reported outcomes** |
| --- | --- | --- | --- | --- | --- | --- | --- | --- |
| Dogru  2023  Turkey | Prospective cohort | Mean: 29.7 | Mean: 28.2 | 32 | 60 | AFI > 24cm | 28-40 weeks | - Caesarean section  - 1m APGAR  - 5m APGAR  - Stillbirth  - NICU admission |
| Pagan  2023  USA | Retrospective cohort | Mean: 29 | Mean: 27 | 249 | 12476 | AFI > 24 cm | n/a | - Caesarean section  - Shoulder dystocia  - PPROM  - PIH/PET  - Preterm birth  - Cord prolapse  - Malpresentation  - NICU admission  - Perinatal mortality |
| Lando  2023  Israel | Retrospective cohort | Mean: 29.5 | Mean: 28.4 | 750 | 7000 | AFI > 24cm or  SDP > 8 cm | n/a | - PPH  - PPROM  - Caesarean section  - Cord prolapse  - 5m APGAR  - Epidural analgesia  - Induction of labour  - Macrosomia  - NICU admission |
| Wax  2022  USA | Retrospective cohort | Mean: 31.5 | Mean: 31.3 | 435 | 435 | AFI > 24 cm | n/a | - Caesarean section  - Cord prolapse  - Perinatal mortality  - 5m APGAR  - Malpresentation  - Macrosomia  - NICU admission |
| Pasquini  2022  Italy | Retrospective cohort | Mean: 35.6 | Mean: 31.8 | 109 | 2550 | AFI > 25 cm or  DVP > 8 cm | n/a | - PPH  - PPROM  - Caesarean section  - Cord prolapse  - Malpresentation  - Induction of labour  - Macrosomia  - 1m APGAR |
| Vanda  2022  Iran | Prospective cohort | Mean: 28.3 | Mean: 27.5 | 90 | 90 | AFI > 24cm or  DVP >8cm | n/a | - PPH  - Caesarean section  - Perinatal mortality  - Macrosomia  - NICU admission |
| Akkaya  2020  Turkey | Prospective cohort | Mean: 27.5 | Mean: 28.5 | 120 | 53 | AFI > 25cm | > 13 weeks | - Caesarean section  - 1m APGAR  - 5m APGAR |
| Cen  2020  China | Prospective cohort | Mean: 31.1 | Mean: 30.6 | 20 | 20 | AFI > 24cm or  SDP > 8 cm | n/a | - Caesarean section |
| Hadar  2020  Israel | Retrospective cohort | Mean: 29.1 | Mean: 27.8 | 5684 | 186195 | AFI > 25cm or  DVP > 8cm | > 20 weeks | - Caesarean section |
| Pariente  2020  Israel | Retrospective cohort | Mean: 29.1 | Mean: 27.8 | 5684 | 186,195 | AFI > 25cm or  DVP > 8cm | > 20 weeks | - Labour induction  - Macrosomia  - Low birthweight |
| Amitai  2019  Israel | Retrospective cohort | Mean: 29.1 | Mean: 27.8 | 5684 | 186,195 | AFI > 25cm or  DVP > 8cm | > 20 weeks | - Caesarean section  - Preterm birth  - 1m APGAR  - 5m APGAR  - Macrosomia |
| Berezowsky  2019  Israel | Retrospective cohort | Median: 32 | Median: 31 | 292 | 29682 | AFI > 25 cm | 28-32 weeks | - PPH  - PIH/PET  - Preterm birth  - 1m APGAR  - 5m APGAR  - Malpresentation  - Breech presentation  - Epidural analgesia  - Labour induction  - Shoulder dystocia  - Macrosomia  - Stillbirth  - Abruption  - NICU admission  - Perinatal mortality |
| Asadi  2018  Iran | Retrospective cohort | Mean: 28.7 | Mean: 26.8 | 114 | 114 | AFI > 24 cm with no defined caused | n/a | - PPH  - Caesarean section  - Abruption  - Perinatal mortality  - Preterm birth  - Stillbirth  - 1m APGAR  - 5m APGAR  - Malpresentation  - Low birth weight  - Macrosomia  - NICU admission |
| Crimmins  2018  USA | Retrospective cohort | Mean: 28.3 | Mean: 24.8 | 282 | 663 | AFI > 25 cm or  MVP ≥ 8cm | > 24 weeks | - PPH  - PIH/PET  - Caesarean section  - Labour induction  - Shoulder dystocia  - LGA- Macrosomia  - NICU admission |
| Depla  2017  Netherlands | Retrospective cohort | n/a | n/a | 15 | 215 | AFI > 95th percentile or  DVP ≥ 8 cm. | 20-36 weeks | - Caesarean section  - Perinatal mortality  - Preterm birth  - Stillbirth  - NICU admission |
| Karahanoglu  2017  Turkey | Retrospective cohort | Mean: 25.8 | Mean: 27.1 | 182 | 392 | AFI > 24 cm | 37-41 weeks | - Malpresentation |
| Khan and Donelly  2017  Ireland | Retrospective cohort | Median: 32 | Median: 33 | 144 | 144 | AFI ≥ 25 cm or  DVP ≥ 8 | 16-41 weeks | - Caesarean section  - Perinatal mortality  - Preterm birth  - Stillbirth  - 1m APGAR  - 5m APGAR  - Low birth weight  - Macrosomia  - NICU admission |
| Luo  2017  China | Retrospective cohort | Mean: 28.5 | Mean: 28 | 307 | 86610 | AFI > 25 cm | n/a | - PPH  - Caesarean section  - Perinatal mortality  - 1m APGAR  - 5m APGAR  - Malpresentation  - Low birth weight  - Macrosomia  - NICU admission |
| Zeino  2017  France | Retrospective cohort | Mean: 29.7 | Mean: 30.5 | 94 | 188 | AFI ≥ 25 cm or  SDP > 10cm | > 13 weeks | - Caesarean section  - Labour induction  - NICU admission |
| Ersoy  2016  Turkey | Prospective cohort | Mean: 25.3 | Mean: 26.5 | 23 | 36 | AFI > 24 cm | n/a | - NICU admission |
| Karahanoglu  2016  Turkey | Retrospective cohort | Mean: 28.5  Median: 26  Range: 16-46 | Mean: 26.5  Median: 26  Range: 16-45 | 207 | 336 | AFI > 24 cm | n/a | - Caesarean section  - Preterm birth  - 5m APGAR  - Cephalopelvic dis  - Malpresentation  - Labour induction  - Macrosomia  - NICU admission |
| Liu  2016  China | Retrospective cohort | n/a | n/a | 50 | 80 | AFI > 25cm or  DVP ≥ 8 cm | n/a | - Preterm birth  - Labour induction  - Macrosomia |
| Wiegand  2016  USA | Retrospective cohort | Mean: 29.5 | Mean: 27.8 | 348 | 10188 | AFI > 24 cm | ≥24 weeks | - PPH  - PPROM  - PIH/PET  - Caesarean section  - Cord prolapse  - Abruption  - Perinatal mortality  - Preterm birth  - Stillbirth  - 5m APGAR  - Macrosomia  - NICU admission |
| Aviram  2015  Israel | Retrospective cohort | Mean: 31.3 | Mean: 30.5 | 215 | 31161 | AFI > 25 cm | ≥ 34 weeks | - PPH  - PIH/PET  - Caesarean section  - Cord prolapse  - Abruption  - Perinatal mortality  - Preterm birth  - Stillbirth  - 1m APGAR  - 5m APGAR  - Epidural analgesia  - Labour induction  - Shoulder dystocia  - NICU admission |
| Cho  2015  South Korea | Case control study | Mean: 30.1 | Mean: 30.6 | 20 | 10 | AFI ≥ 24 cm | n/a | - Caesarean section |
| Lallar  2015  India | Case control study | Mean: 28.7  Range: 18-38 | Mean: 27.8  Range: 18-38 | 500 | 500 | AFI ≥ 24 cm | 28-36 weeks | - PPH  - PPROM  - PIH/PET  - Shoulder dystocia  - Caesarean section  - Abruption  - Perinatal mortality  - Preterm birth  - Stillbirth  - Cephalopelvic disproportion  - Transverse lie  - Breech  - Malpresentation  - Macrosomia  - NICU admission |
| Stanescu  2015  Romania | Retrospective cohort | n/a | n/a | 295 | 24214 | AFI > 24 cm | 28-38 weeks | - PPROM  - Caesarean section  - Cord prolapse  - Abruption  - Perinatal mortality  - Preterm birth  - 5m APGAR  - Malpresentation  - Low birth weight  - Macrosomia  - NICU |
| Akin  2013  Turkey | Prospective cohort | Mean: 25.6 | Mean: 24.5 | 29 | 50 | AFI > 25cm | 35-40 weeks | - Caesarean section  - Cord prolapse  - Cephalopelvic disproportion |
| Taskin  2013  Turkey | Retrospective cohort | Mean: 29.7 | Mean: 29 | 59 | 101 | AFI > 20 cm | ≥ 20 weeks | - Caesarean section  - Cord prolapse  - Perinatal mortality  - Preterm birth  - 1m APGAR  - 5m APGAR  - Cephalopelvic disproportion  - Transverse lie  - Malpresentation  - Low birth weight  - Macrosomia  - NICU admission |
| Zhu  2010  China | Prospective cohort | Mean: 29.6 | Mean: 26.7 | 21 | 30 | AFV > 2000 ml | n/a | - Caesarean section |
| Porter  2004  USA | Prospective cohort | n/a | Mean: 24.4 | 16 | 11 | AFI > 24cm | 24-28 weeks | - Shoulder dystocia  - Macrosomia  - NICU admission |
| Hershkovitz  2002  Israel | Prospective cohort | Mean: 27.9 | Mean: 28.5 | 72 | 72 | AFI > 24cm | n/a | - PIH/PET  - Caesarean section  - 5m APGAR  - NICU admission |
| Hershkovitz  2001  Israel | Prospective cohort | n/a | n/a | 113 | 113 | AFI > 24cm | n/a | - PIH/PET  - Caesarean section |
| Panting-Kemp  1999  USA | Prospective cohort | n/a | n/a | 151 | 302 | AFI > 24 cm | 20-40 weeks | - Caesarean section  - Perinatal mortality  - Preterm birth  -Stillbirth  - Cephalopelvic disproportion  - 5m APGAR  - Malpresentation  - Low birth weight  - Macrosomia  - NICU admission |
| Maymon  1997  Israel | Cross-sectional study | Mean: 30.7 | Mean: 28.1 | 1211 | 59491 | AFI > 25 cm / vertical pocket > or equal to 8 cm | > 28 weeks | - PIH/PET  - Caesarean section  - Cord prolapse  - Placental abruption  - Perinatal mortality  - Stillbirth  - 5m APGAR  - Malpresentation  - Labour induction |
| Glantz  1997  USA | Prospective cohort | Mean: 23 | Mean: 23.9 | 50 | 85 | SDP > 6 cm | 14-27 weeks | - PPH  - PIH/PET  - Caesarean section  - Placental abruption  - Perinatal mortality  - Stillbirth  - Preterm labour  - Malpresentation |
| Sohaey  1994  USA | Retrospective cohort | n/a | n/a | 99 | 99 | AFI ≥ 24 cm | n/a | - Perinatal mortality  - Stillbirth |
| Smith  1992  USA | n/a | n/a | n/a | 97 | 462 | AFI > 24 cm | >26 weeks | - PPH  - Caesarean section  - Cord prolapse  - Placental abruption  - Perinatal mortality  - Preterm birth  - Stillbirth  - 1m APGAR  - 5m APGAR  - Malpresentation  - Macrosomia |

1m: 1 minute / 5m: 5 minute

AFI: amniotic fluid volume

APGAR: appearance, pulse, grimace, activity, and respiration score

DVP: deepest vertical pocket

IP: idiopathic polyhydramnios

n/a: not available

NICU: neonatal intensive care unit

PIH/PET: pregnancy induced hypertension/pre-eclamptic toxaemia

PPH: post-partum haemorrhage

PPROM: preterm premature rapture of membranes

SDP: single deepest pool,

**Table S2.** Inclusion and exclusion criteria of the included studies.

| **Author**  **Year**  **Country** | **Definition of Idiopathic polyhydramnios**  **(cases)** | **Inclusion criteria** | **Exclusion criteria** |
| --- | --- | --- | --- |
| Dogru  2023 | AFI > 24cm | - Singleton pregnancies | - Twin pregnancies - Structural and chromosomal anomalies - Fetal anaemia - Fetal growth restriction - PET - Chronic drug addiction - Maternal or fetal infection - Gestational diabetes - PPROM |
| Pagan  2023 | AFI > 24 cm | - Singleton pregnancies | - Oligohydramnios - Structural and chromosomal anomalies - Twin pregnancies - Placenta tumours - Gestational diabetes - Maternal or fetal infection |
| Lando  2023 | AFI > 24cm or  SDP > 8 cm | - Singleton pregnancies - IP confirmed within 14 days of term delivery - Spontaneous delivery or induction of labour | - Preterm or post-term deliveries - Structural and chromosomal anomalies - Fetal growth restriction - Multiple pregnancies - Caesarean deliveries - Gestational diabetes |
| Wax  2022 | AFI > 24 cm | - Singleton pregnancies - Patients older than 18 years | - Structural and chromosomal anomalies - Placental tumours - Fetal infection - Gestational diabetes |
| Pasquini  2022 | AFI > 25 cm or  DVP > 8 cm | - Singleton pregnancies | - Structural and chromosomal anomalies - Maternal or fetal infection - Gestational diabetes |
| Vanda  2022 | AFI > 24cm or  DVP >8cm | - Singleton pregnancies | - Structural and chromosomal anomalies - Gestational diabetes |
| Akkaya  2020 | AFI > 25cm | - Singleton pregnancies - Delivery between 29-41 weeks of gestation | - Chronical maternal disease - Fetal abnormalities - Delivery withing three days of the ultrasound examination - Gestational diabetes - Gestational hypertensive disorders - Isoimmunization - Maternal or fetal infection - Positive OGTT |
| Cen  2020 | AFI > 24cm  SDP > 8 cm | - Singleton pregnancies - Delivery between 37-40 weeks of gestation | - Fetal or placental abnormalities - Fetal or maternal haemorrhage - Fetal infections - Gestational diabetes - Isoimmunization |
| Hadar  2020 | AFI > 25cm  DVP > 8 cm | - Singleton pregnancies | - Fetal and chromosomal abnormalities - Gestational or pre-gestational diabetes - Gestational hypertension - Intra-uterine growth restriction - Isoimmunizations - Lack of prenatal care - Placental abruption - Multiple gestations - Premature rupture of membranes |
| Pariente  2020 | AFI ≥ 25 cm | - Singleton pregnancies | - Chromosomal abnormalities - Congenital malformations - Gestational or pre-gestational diabetes - Gestational hypertensive disorders - Isoimmunisation - Lack of prenatal care - Multiple gestations - Placental abruption - Premature rupture of membranes - Perinatal mortality |
| Amitai  2019 | AFI >24 cm  MVP > 8 cm | - Singleton pregnancies | - Chromosomal abnormalities - Congenital malformations - Gestational or pre-gestational diabetes - Gestational hypertensive disorders - Intra-uterine growth restriction - Isoimmunization - Lack of prenatal care - Perinatal mortality - Premature rupture of membranes |
| Berezowsky  2019 | AFI > 25 cm | - Singleton pregnancies | - Gestational or pre-gestational diabetes - Home or car delivery - Incomplete data - Intra-uterine growth restriction - Premature rupture of membranes - Suspected cardiac, gastrointestinal, renal or central nervous system anomalies, - Termination of pregnancy |
| Asadi  2018 | AFI > 24 cm | - Singleton pregnancies | - Abnormal placentation - Chromosomal abnormalities - Fetal abnormalities - Gestational diabetes - Isoimmunization - Multiple gestations |
| Crimmins  2018 | AFI >25 cm  MVP ≥ 8 cm | - Singleton pregnancies - Non-anomalous pregnancies - OGCT (<130mg/dL) at 24-28 weeks of gestation | - Chromosomal abnormalities - Fetal infection - Incomplete glucose screening during pregnancy - Isoimmunization - Multiple gestations |
| Depla  2017 | AFI > 95th percentile DVP ≥ 8 cm | - Singleton pregnancies - Prenatally diagnosed with oral cleft palate | - Gestational diabetes - Macrosomia - TORCH infection |
| Karahonoglu  2017 | AFI > 24 cm | - Singleton pregnancies | - Fetal abnormalities - Gestational diabetes - Gestational hypertensive disorders - Placental insufficiency - Renal disorders |
| Khan  2017 | DVP ≥ 8  AFI ≥ 25 cm | - Singleton pregnancies | - Fetal abnormalities - Gestational diabetes - Isoimmunisation - Multiple gestations - TORCH infection |
| Luo  2017 | AFI >25 cm | - Delivery between 37 and >41 weeks of gestation | - Congenital abnormalities - Gestational diabetes - Gestational hypertensive disorders - Multiple gestations - Placental abnormalities |
| Zeino  2017 | AFI ≥ 25 cm  SDP > 10 cm | - Singleton pregnancies at second or third trimester of gestation - Delivery at >37 weeks of gestation - Cephalic presentation | - Breech or transverse positions - Gestational diabetes - Isoimmunization - Oesophageal atresia, Perre robin sequence, cardiac malformations - Positive cytomegalovirus, syphilis, Epstein barr, B19, toxoplasmosis - Premature deliveries - Previous caesarean section, |
| Ersoy  2016 | AFI > 24 cm | - Singleton pregnancies at >28 weeks of gestation | - Doppler waveform abnormalities in the uterine, umbilical and middle cerebral arteries - Fetal anaemia - Fetal abnormalities - Intra-uterine growth restriction - Maternal systemic disease - Rupture of the membranes - Use of various drugs/substances that are likely to affect the circulatory system of the fetus (painkillers, alcohol, tobacco). |
| Karahonoglu  2016 | AFI > 24 cm | - Singleton pregnancies | - Abnormal fetal karyotype - Congenital anomalies detected antenatally - Gestational diabetes |
| Liu  2016 | AFI > 25 cm  DVP ≥ 8 cm | - Singleton pregnancies - Prenatal scan | - Family history of systemic disease - Family history of congenital malformations - Exposure to alcohol, tobacco smoke, irradiation or infectious diseases during pregnancy - Nonconsanguineous parents |
| Wiegand  2016 | AFI > 24 cm | - Delivery between 24 and 42 weeks of gestation - Prenatal ultrasound | - Fetal abnormalities - Gestational or pre-gestational diabetes - Isoimmunization - Multiple gestations |
| Aviram  2015 | AFI > 25 cm | - Gestations ≥ 34 weeks | - Fetal or chromosomal abnormalities - Gestational or pre-gestational diabetes mellitus - Home or car delivery - Incomplete antepartum surveillance - Intra-uterine growth restriction - Intra-uterine infection - Missing data - Prelabour caesarean delivery - Previously diagnosed polyhydramnios with normal amniotic fluid index at admission for labour - Termination of pregnancy |
| Cho  2015 | AFI ≥ 24 cm | - Singleton pregnancies | - Congenital abnormalities - Fetal infection - Gestational diabetes - Gestational hypertensive disorders - Isoimmunization - Maternal vascular disease - Multiple gestations - Placental tumours - Previous diagnosis of diabetes |
| Lallar  2015 | AFI ≥ 24 cm | - Singleton pregnancies at second or third trimester of gestation | - Fetal abnormalities - Gestational diabetes - Hydrops - Multiple gestations - Placental abnormalities |
| Stanescu  2015 | AFI > 24 cm | - Singleton pregnancies | - Gestational diabetes - Fetal abnormalities - Fetal anaemia - Fetal or maternal infections - Multiple pregnancies |
| Akin  2013 | AFI > 25 cm | - Singleton pregnancies between 35-40 weeks of gestation. - Normal OGTT - Negative PROM tests | - Fetal abnormalities - History of smoking, alcohol consumption or medication use - Previous caesarean section or uterine surgery |
| Taskin  2013 | AFI > 20 cm | - Singleton pregnancies at >20 weeks of gestation | - Fetal or placental abnormalities - Gestational diabetes - Intra-uterine infections - Isoimmunization - Multiple gestations - Previous diagnosis of diabetes |
| Zhu  2010 | AFV > 2000 ml | - Singleton pregnancies delivered between 37 - 40 weeks gestation - Nulliparous patients with elective caesarean deliveries | - Fetal infections - Fetal or placental abnormalities - Fetal or maternal haemorrhage - Gestational diabetes - Isoimmunization - Previous diagnosis of diabetes |
| Porter  2004 | AFI > 24 cm | - Nulliparous or multiparous pregnant women between 24 and 39 weeks of gestation | - Age <18 years old - Fetal abnormalities - Gestational diabetes - Mental disability - Previous diagnosis of diabetes |
| Hershkovitz  2002 | AFI > 24 cm | - Singleton pregnancies | - Fetal infections - Fetal or placental abnormalities - Gestational diabetes - Isoimmunization - Previous diagnosis of diabetes |
| Hershkovitz  2001 | AFI > 24 cm |  | - Fetal, chromosomal or placental abnormalities - Gestational diabetes - Isoimmunization - Previous diagnosis of diabetes |
| Panting-Kemp  1999 | AFI > 24 cm | - Singleton pregnancies | - Fetal or placental abnormalities - Gestational or pre-gestational diabetes - Isoimmunization - Multiple gestations |
| Maymon  1997 | AFI > 25 cm  vertical pocket ≥ 8 cm | - Singleton gestation - Delivery at >37 weeks of gestation | - Lack of prenatal care - Unknown gestational age - Unreliable menstrual history |
| Glantz  1994 | SDP > 6 cm | - Singleton pregnancies between 14-27 weeks of gestation - Absence of sonographic signs of foetal structural or placental anomalies - Normal OGGT | - Isoimmunization - Multiple gestations |
| Sohaey  1994 | AFI ≥ 24 cm | - Non-diabetic women with a normal result of a diabetes screening examination at 24-26 weeks of gestation | - Fetal abnormalities - Multiple gestations - Unreliable menstrual history |
| Smith  1992 | AFI > 24 cm | - Gestations > 26 weeks | - Fetal abnormalities - Gestational diabetes - Isoimmunization - Maternal symptoms before polyhydramnios - Multiple gestations |

Abbreviations

AFI: amniotic fluid index

DVP: deepest vertical pocket

SDP: single deepest pocket

MVP: maximal vertical pocket

OGTT: oral glucose tolerance test

PROM: preterm rupture of membrane

**Table S3.** Definition of outcomes in the included studies**.**

| **Author**  **Year**  **Country** | **PPH** | **PPROM** | **PIH/PE** | **Perinatal morality** | **Preterm birth** | **5’ APGAR** | **1’ APGAR** | **Preterm labour** | **Shoulder dystocia** |
| --- | --- | --- | --- | --- | --- | --- | --- | --- | --- |
| Dogru,  2023 | **-** | - | - | - | - | - | - | - | - |
| Pagan,  2023 | - | **NA** | **NA** | - | - | - | - | - | **NA** |
| Lando,  2023 | **NA** | - | - | - | - | <7 | - | - | - |
| Wax,  2022 | - | - | - | - | - | <7 | - | - | - |
| Pasquini,  2022 | **NA** | - | - | - | - | - | <7 | - | - |
| Vanda,  2022 | **NA** | - | - | - | - | - | - | - | - |
| Akkaya,  2020 | - | - | - | - | - | <7 | <7 | - | - |
| Cen,  2020 | - | - | - | - | - | - | - | - | - |
| Hadar,  2020 | - | - | - | - | - | - | - | - | - |
| Pariente,  2020 | - | - | - | - | - | - | - | - | - |
| Amitai,  2019 | - | - | - | - | NA | <7 | <7 | - | - |
| Berezowsky, 2019 | NA | - | NA | NA | <37 weeks | NA | NA | - | head-to-shoulder  delivery time exceeded 60 seconds or when ancillary measures were used in order to extract the neonate shoulder |
| Asadi,  2018 | NA | - | - | NA | <37 weeks | <7 | <7 | - | - |
| Crimmins, 2018 | NA | - | NA | - | - | - | - | - | NA |
| Depla,  2017 | - | - | - | Death within 1 week after delivery | <37 weeks | - | - | - | - |
| Karahanoglu, 2017 | - | - | - | - | - | - | - | - | - |
| Khan,  2017 | - | - | - | NA | <37 weeks | <7 | <7 | - | - |
| Luo,  2017 | NA | - | - | Death within 27 days after delivery | - | <7 | <7 | - | - |
| Zeino,  2017 | - | - | - | - | - | - | - | - | - |
| Ersoy,  2016 | - | - | - | - | - | - | - | - | - |
| Karahanoglu, 2016 | - | - | - | - | NA | <7 | - | - | - |
| Liu,  2016 | - | NA | - | - | <37 weeks | - | - | - | - |
| Wiegand,  2016 | >1L | <37 weeks | Criteria based on American Congress of Obstetricians and Gynaecologists 2013 | Death within 28 days after delivery | <37 weeks | <7 | - | - | - |
| Aviram,  2015 | NA |  | Criteria based on International Society for the Study of Hypertension in Pregnancy guidelines 2001 | NA | - | <7 | <7 | - | head-to shoulder delivery time exceeded 60 seconds or when ancillary measures were used to extract the neonate shoulder |
| Cho,  2015 | - | - | - | - | - | - | - | - | - |
| Lallar,  2015 | NA | NA | NA | Death within 30 days after delivery | NA | - | - | NA | - |
| Stanescu,  2015 | - | NA | - | NA | <37 weeks | <7 | - | - | - |
| Akin,  2013 | - | - | - | - | - | - | - | - | - |
| Taskin,  2013 | - | - | - | Death within 7 days and 365 days after delivery | <37 weeks | <7 | <7 | - | - |
| Zhu,  2010 | - | - | - | - | - | - | - | - | - |
| Porter,  2004 | - | - | - | - | - | - | - | - | NA |
| Hershkovitz,  2002 | - | - | NA | - | - | <7 | - | - | - |
| Hershkovitz, 2001 | - | - | NA | - | - | <7 | - | - | - |
| Panting-Kemp,  1999 | - | - | - | Death >20 weeks of gestation or before discharge from hospital | <37 weeks | <7 | - | - | - |
| Maymon,  1997 | - | - | NA | Death within 28 days after delivery | - | <7 | - | - | - |
| Glantz,  1994 | NA | NA | NA | NA | NA | NA | - | NA | - |
| Sohaey,  1994 | - | - | - | NA | - | - | - | - | NA |
| Smith,  1992 | NA | NA | - | - | <37 weeks | <7 | - | <37 weeks |  |

Abbreviations

1m: 1 minute / 5m: 5 minute

APGAR: appearance, pulse, grimace, activity, and respiration score

NA: not available

PIH/PE: pregnancy induced hypertension/pre-eclampsia

PPH: post-partum haemorrhage

PPROM: preterm premature rapture of membranes

**Table S4.** Risk of bias assessment for the included studies based on ROBINS-E tool*.

| **Author, year** | **Domain 1** | **Domain 2** | **Domain 3** | **Domain 4** | **Domain 5** | **Domain 6** | **Domain 7** | **Total** |
| --- | --- | --- | --- | --- | --- | --- | --- | --- |
| Dogru, 2023 | Low | Low | Some concerns | Low | Low | Low | Low | Some concerns |
| Pagan, 2023 | Low | Low | Some concerns | Low | Low | Low | Low | Some concerns |
| Lando, 2023 | Low | Low | Some concerns | Low | Low | Low | Low | Some concerns |
| Wax, 2022 | Low | Low | Some concerns | Low | Low | Low | Some concerns | Some concerns |
| Pasquini, 2022 | Low | Low | Some concerns | Low | Low | Low | Some concerns | Some concerns |
| Vanda, 2022 | Low | Low | Low | Low | Low | Low | Low | Low |
| Akkaya, 2020 | Low | Low | Some concerns | Low | Low | Low | Low | Some concerns |
| Cen, 2020 | Low | Low | Some concerns | Low | Low | Low | Low | Some concerns |
| Hadar, 2020 | Low | Low | Some concerns | Low | Low | Low | Low | Some concerns |
| Pariente, 2020 | Low | Low | Some concerns | Low | Low | Low | Low | Some concerns |
| Amitai, 2019 | Low | Low | Some concerns | Low | Low | Low | Low | Some concerns |
| Berezowsky, 2019 | Low | Low | Some concerns | Low | Low | Low | Low | Some concerns |
| Asadi, 2018 | Low | Low | Some concerns | Low | Low | Low | Low | Some concerns |
| Crimmins, 2018 | Low | Low | Some concerns | Low | Low | Low | Some concerns | Some concerns |
| Depla, 2017 | Some concerns | Low | Some concerns | Low | Low | Low | Low | Some concerns |
| Karahanoglu, 2017 | Low | Low | Some concerns | Low | Low | Low | Low | Some concerns |
| Khan, 2017 | Low | Low | Low | Low | Low | Low | Low | Some concerns |
| Luo, 2017 | Low | Low | Some concerns | Low | Low | Low | Low | Some concerns |
| Zeino, 2017 | Low | Low | Some concerns | Low | Low | Low | Low | Some concerns |
| Ersoy, 2016 | Low | Low | Low | Low | Low | Low | Some concerns | Some concerns |
| Karahanoglu, 2016 | Low | Low | Some concerns | Low | Low | Low | Low | Some concerns |
| Liu, 2016 | Low | Low | Some concerns | Low | Low | Low | Low | Some concerns |
| Wiegand, 2016 | Low | Low | Some concerns | Low | Low | Low | Low | Some concerns |
| Aviram, 2015 | Low | Low | Some concerns | Low | Low | Low | Low | Some concerns |
| Cho, 2015 | Low | Low | Some concerns | Low | Low | Low | Low | Some concerns |
| Lallar, 2015 | Some concerns | Low | Some concerns | Low | Low | Low | Low | Some concerns |
| Stanescu, 2015 | Low | Low | Some concerns | Low | Low | Low | Low | Some concerns |
| Akin, 2013 | High | Low | Low | Low | Low | Low | Low | High |
| Taskin, 2013 | Low | Low | Some concerns | Low | Low | Low | Low | Some concerns |
| Zhu, 2010 | Low | Low | Some concerns | Low | Low | Low | Low | Some concerns |
| Porter, 2004 | Low | Low | Some concerns | Low | Low | Low | Low | Some concerns |
| Hershkovitz, 2002 | Low | Low | Low | Low | Low | Low | Low | Low |
| Hershkovitz, 2001 | Low | Low | Low | Low | Low | Low | Low | Low |
| Panting-Kemp, 1999 | Some concerns | Low | Low | Low | Low | Low | Low | Low |
| Maymon, 1997 | Low | Low | Low | Low | Low | Low | Low | Low |
| Glantz, 1994 | High | Low | Low | Low | Low | Low | Low | High |
| Sohaey, 1994 | Low | Low | Low | Low | Low | Low | Low | Low |
| Smith, 1992 | Low | Low | Low | Low | Low | Low | Low | Low |

* The Risk Of Bias In Non-randomized Studies - of Exposure (ROBINS-E) is a tool that provides a systematic approach to assessing the risk of bias in observational epidemiological studies. ROBINS-E contributes to a thorough examination of the strength of evidence about the presence of, and/or nature of, a potential effect of an exposure on an outcome. A key feature of the ROBINS-E approach is the specification, for each study, of the causal effect estimated by the result under consideration. ROBINS-E is based on 7 domains which assess biases related to confounders, selection of participants, definition of exposure, missing data, measurement of outcome and selection of reporting results.

**Table S5.** Quality rating for the analyses of outcomes based on GRADE.

| **Certainty assessment** | | | | | | | **Summary of findings** | | | | |
| --- | --- | --- | --- | --- | --- | --- | --- | --- | --- | --- | --- |
| **Studies**  **(participants)** | **Risk of bias** | **Inconsistency *** | **Indirectness **** | **Imprecision***** | **Publication bias** | **Overall certainty of evidence †** | **Study event rates (%)** | | **RR**  **(95% CI)** | **Anticipated absolute effects** | |
|  |  |  |  |  |  |  | **Control group** | **IP group** |  | **Risk with control** | **Risk difference with IP** |
| **Preterm birth** | | | | | | | | | | | |
| 304563 (15 observational studies) | not serious | Serious ^a^ | not serious | not serious | none | ⨁◯◯◯ Very low | 18830/296171 (6.3%) | 901/8392  (10.7%) | **1.96** (1.35 to 2.86) | 59 per 1,000 | **64 more per 1,000** (from 21 more to 129 more) |
| **PPROM** | | | | | | | | | | | |
| 59176 (6 observational studies) | not serious | Serious ^a^ | not serious | not serious | none | ⨁◯◯◯ Very low | 2775/56928 (4.8%) | 151/2248  (6.7%) | **1.27** (0.50 to 3.25) | 44 per 1,000 | **40 more per 1,000** (from 14 fewer to 192 more) |
| **PIH/PET** | | | | | | | | | | | |
| 147852 (11 observational studies) | not serious | not serious | not serious | not serious | publication bias strongly suspected ^b^ | ⨁◯◯◯ Very low | 4439/144488 (3%) | 133/3364  (3.9%) | **1.01** (0.81 to 1.28) | 16 per 1,000 | **0 fewer per 1,000** (from 4 fewer to 7 more) |
| **Epidural analgesia** | | | | | | | | | | | |
| 69100 (3 observational studies) | not serious | not serious | not serious | not serious | none | ⨁⨁◯◯ Low | 43410/67843 (63.9%) | 880/1257 (70.0%) | **1.08** (0.98 to 1.18) | 633 per 1,000 | **82 more per 1,000** (from 44 more to 120 more) |
| **Labour induction** | | | | | | | | | | | |
| 326212 (10 observational studies) | not serious | Serious ^a^ | not serious | not serious | none | ⨁◯◯◯ Very low | 49283/317346 (17.6%) | 2387/8866 (28.9%) | **1.53** (1.18 to 2.00) | 154 per 1,000 | **109 more per 1,000** (from 38 more to 206 more) |
| **Caesarean section** | | | | | | | | | | | |
| 465744 (31 observational studies) | not serious | Serious ^a^ | not serious | not serious | none | ⨁◯◯◯ Very low | 95945/453610 (21.1%) | 3301/12134 (27.2%) | **1.60** (1.39 to 1.84) | 209 per 1,000 | **128 more per 1,000** (from 77 more to 188 more) |
| **Cord prolapse** | | | | | | | | | | | |
| 151925 (11 observational studies) | not serious | not serious | not serious | not serious | none | ⨁⨁◯◯ Low | 343/148128 (0.2%) | 43/3797  (1.1%) | **6.5** (4.68 to 9.04) | 3 per 1,000 | **14 more per 1,000** (from 9 more to 22 more) |
| **Placental abruption** | | | | | | | | | | | |
| 159019 (9 observational studies) | not serious | not serious | not serious | not serious | none | ⨁⨁◯◯ Low | 540/155897 (0.3%) | 64/3122  (2.0%) | **3.20** (2.20 to 4.65) | 3 per 1,000 | **8 more per 1,000** (from 4 more to 13 more) |
| **Malpresentation** | | | | | | | | | | | |
| 212008 (15 observational studies) | not serious | not serious | not serious | not serious | none | ⨁⨁◯◯ Low | 7793/2077509 (3.7%) | 294/4258 (6.9%) | **1.82** (1.42 to 2.35) | 36 per 1,000 | **39 more per 1,000** (from 20 more to 64 more) |
| **Cephalopelvic disproportion** | | | | | | | | | | | |
| 2235 (5 observational studies) | not serious | Serious ^a^ | not serious | not serious | none | ⨁◯◯◯ Very low | 26/1289  (2.0%) | 44/946  (4.7%) | **2.43** (1.02 to 5.84) | 20 per 1,000 | **29 more per 1,000** (from 0 fewer to 98 more) |
| **Transverse lie** | | | | | | | | | | | |
| 1160 (2 observational studies) | not serious | not serious | not serious | not serious | none | ⨁⨁◯◯ Low | 13/601  (2.2%) | 33/559  (5.9%) | **2.64** (1.29 to 5.42) | 22 per 1,000 | **35 more per 1,000** (from 6 more to 96 more) |
| **Breech presentation** | | | | | | | | | | | |
| 30974 (2 observational studies) | not serious | Serious ^a^ | not serious | not serious | none | ⨁◯◯◯ Very low | 1083/30182 (3.6%) | 27/792  (3.4%) | **1.34** (0.52 to 3.46) | 36 per 1,000 | **12 more per 1,000** (from 17 fewer to 88 more) |
| **PPH** | | | | | | | | | | | |
| 172249 (12 observational studies) | not serious | Serious ^a^ | not serious | not serious | none | ⨁◯◯◯ Very low | 5158/1690955 (3.0%) | 220/3154 (6.9%) | **1.98** (1.22 to 3.22) | 26 per 1,000 | **38 more per 1,000** (from 10 more to 91 more) |
| **Perinatal mortality** | | | | | | | | | | | |
| 261038 (18 observational studies) | not serious | not serious | not serious | not serious | none | ⨁⨁◯◯ Low | 723/256367 (0.3%) | 187/4671 (3.4%) | **4.75** (2.67 to 8.48) | 3 per 1,000 | **13 more per 1,000** (from 6 more to 26 more) |
| **Stillbirth** | | | | | | | | | | | |
| 135771 (13 observational studies) | not serious | not serious | not serious | not serious | none | ⨁⨁◯◯ Low | 221/132503 (0.1%) | 74/3268  (2.2%) | **4.75** (2.54 to 8.86) | 2 per 1,000 | **6 more per 1,000** (from 3 more to 13 more) |
| **Shoulder dystocia** | | | | | | | | | | | |
| 75047 (5 observational studies) | not serious | Serious ^a^ | not serious | not serious | none | ⨁◯◯◯ Very low | 240/73993 (0.3%) | 21/1054  (2.0%) | **3.52** (2.08 to 5.96) | 3 per 1,000 | **8 more per 1,000** (from 3 more to 17 more) |
| **Low birth weight** | | | | | | | | | | | |
| 304434 (7 observational studies) | not serious | Serious ^a^ | not serious | not serious | none | ⨁◯◯◯ Very low | 12225/297680 (4.1%) | 267/6754 (4.0%) | **1.21** (0.74 to 1.98) | 41 per 1,000 | **9 more per 1,000** (from 11 fewer to 40 more) |
| **Macrosomia** | | | | | | | | | | | |
| 546459 (20 observational studies) | not serious | not serious | not serious | not serious | none | ⨁⨁◯◯ Low | 27506/530873 (5.1%) | 2001/15586 (12.8%) | **2.68** (2.43 to 2.95) | 51 per 1,000 | **82 more per 1,000** (from 70 more to 95 more) |
| **NICU admission** | | | | | | | | | | | |
| 209409 (22 observational studies) | not serious | Serious ^a^ | not serious | not serious | none | ⨁◯◯◯ Very low | 20863/204719 (10.1%) | 672/4690 (16.3%) | **1.62** (1.11 to 2.37) | 103 per 1,000 | **68 more per 1,000** (from 3 fewer to 189 more) |
| **Abnormal 1m APGAR** | | | | | | | | | | | |
| 344214 (10 observational studies) | not serious | Serious ^a^ | not serious | not serious | none | ⨁◯◯◯ Very low | 10893/337073 (3.2%) | 492/7141 (6.9%) | **2.14** (1.48 to 3.10) | 32 per 1,000 | **42 more per 1,000** (from 18 more to 79 more) |
| **Abnormal 5m APGAR** | | | | | | | | | | | |
| 447062 (17 observational studies) | not serious | Serious ^a^ | not serious | serious | none | ⨁◯◯◯ Very low | 5785/436561 (1.3%) | 209/10501 (2.0%) | **3.00** (1.23 to 7.35) | 13 per 1,000 | **29 more per 1,000** (from 2 more to 105 more) |

* Inconsistency: similarity of point estimates, extent of overlap of confidence intervals, and statistical criteria including tests of heterogeneity and I^2^

** Indirectness: differences in population, intervention and outcome and indirect comparisons

*** Imprecision: examination of 95% CIs and optimal information size

**†** For the aforementioned outcomes plausible residual confounding would potentially suggest spurious effect.

**GRADE Working Group grades of evidence definitions**

High quality: Confidence that the true effect lies close to that of the estimate of the effect

Moderate quality: Moderate confidence in the effect estimate: The true effect is likely to be close to the estimate of the effect, but there is a possibility that it is substantially different
Low quality: Limited confidence in the effect estimate: The true effect may be substantially different from the estimate of the effect

Very low quality: Very little confidence in the effect estimate: The true effect is likely to be substantially different from the estimate of effect

**Table S6.** Sensitivity analysis for the primary outcomes.

|  | **No of studies**  **(references)** | **Total number of participants** | **Intervention Group**  **Events per total**  **n/N (%)** | **Comparison group**  **Events per total**  **n/N (%)** | **RR (95% CI)**  **[Random effect model - Inverse variance method]** | **I^2^** | **t^2^** |
| --- | --- | --- | --- | --- | --- | --- | --- |
| **PRETERM BIRTH** | | | | | | | |
| Excluding studies with serious or critical risk of bias | 14 | 291838 | 849/8143  (10.4%) | 16695/283695  (5.9%) | 2.08 [1.35, 3.20] | 93% | 0.53 |
| Using fixed effect meta-analysis model | 14 | 291838 | 849/8143  (10.4%) | 16695/283695  (5.9%) | 1.58 [1.46, 1.71] | 93% | n/a |
| **PIH/PET** | | | | | | | |
| Excluding studies with serious or critical risk of bias | 8 | 134903 | 85/3033  (2.8%) | 2109/131870  (1.6%) | 1.00 [0.69, 1.44] | 39% | 0.10 |
| Using fixed effect meta-analysis model | 9 | 135038 | 88/3083  (2.9%) | 2114/131955  (1.6%) | 1.01 [0.79, 1.29] | 31% | n/a |
| **LABOUR INDUCTION** | | | | | | | |
| Excluding studies with serious or critical risk of bias | 8 | 315803 | 2223/8007  (27.8%) | 47382/307796  (15.4%) | 1.71 [1.25, 2.34] | 95% | 0.16 |
| Using fixed effect meta-analysis model | 8 | 315803 | 2223/8007  (27.8%) | 47382/307796  (15.4%) | 1.54 [1.48, 1.59] | 95% | n/a |
| **CAESAREAN SECTION** | | | | | | | |
| Excluding studies with serious or critical risk of bias | 23 | 441245 | 2877/10381  (27.7%) | 90242/430864  (30.0%) | 1.63 [1.37, 1.93] | 96% | 0.14 |
| Using fixed effect meta-analysis model | 25 | 441459 | 2894/10460  (27.7%) | 90263/430999  (21.0%) | 1.75 [1.69, 1.81] | 96% | n/a |
| Excluding studies with statistically significant differences in birthweight. | 14 | 94578 | 821/2455  (33.4%) | 7045/92123  (7.6%) | 1.91 [1.42, 2.56] | 96% | 0.26 |
| Excluding studies with a mean birthweight more than 3.5kg | 23 | 441098 | 2854/10337  (27.6%) | 90245/430761  (21.0%) | 1.56 [1.32, 1.84] | 96% | 0.13 |
| **PLACENTAL ABRUPTION** | | | | | | | |
| Excluding studies with serious or critical risk of bias | 8 | 158884 | 63/3072  (2.1%) | 540/155812  (0.3%) | 3.13 [2.05, 4.78] | 15% | 0.05 |
| Using fixed effect meta-analysis model | 9 | 159019 | 64/3122  (2.0%) | 540/155897  (0.3%) | 3.20 [2.19, 4.68] | 2% | n/a |
| **MALPRESENTATION** | | | | | | | |
| Excluding studies with serious or critical risk of bias | 11 | 205619 | 264/3415  (7.7%) | 7382/202204  (3.7%) | 2.07 [1.55, 2.77] | 61% | 0.11 |
| Using fixed effect meta-analysis model | 12 | 205754 | 265/3465  (7.6%) | 7383/202289  (3.6%) | 2.11 [1.83, 2.43] | 57% | n/a |
| **PPH** | | | | | | | |
| Excluding studies with serious or critical risk of bias | 8 | 161525 | 124/2155  (5.8%) | 4117/159370  (2.6%) | 2.53 [1.37, 4.69] | 82% | 0.54 |
| Using fixed effect meta-analysis model | 9 | 161660 | 125/2205  (5.7%) | 4118/159455  (2.6%) | 2.09 [1.68, 2.62] | 79% | n/a |
| Excluding studies with statistically significant differences in birthweight. | 3 | 31739 | 11/379  (2.9%) | 689/31360  (2.2%) | 1.46 [0.70, 3.06] | 0% | 0.00 |
| Excluding studies with a mean birthweight more than 3.5kg | 9 | 161660 | 125/2205  (5.7%) | 4118/159455  (2.6%) | 2.49 [1.38, 4.52] | 79% | 0.52 |
| **PERINATAL MORTALITY** | | | | | | | |
| Excluding studies with serious or critical risk of bias | 14 | 247130 | 180/3847  (4.7%) | 681/243283  (0.3%) | 5.35 [2.78, 10.29] | 44% | 0.42 |
| Using fixed effect meta-analysis model | 15 | 247265 | 182/3897  (4.7%) | 681/243368  (0.3%) | 8.18 [5.87, 11.40] | 48% | n/a |
| **STILLBIRTH** | | | | | | | |
| Excluding studies with serious or critical risk of bias | 11 | 135544 | 72/3186  (2.3%) | 221/132358  (0.2%) | 4.71 [2.34, 9.48] | 18% | 0.20 |
| Using fixed effect meta-analysis model | 12 | 135679 | 74/3236  (2.3%) | 221/132443  (0.2%) | 7.78 [4.36, 13.89] | 35% | n/a |
| **Macrosomia** | | | | | | | |
| Excluding studies with serious or critical risk of bias | 16 | 540000 | 1800/14202  (12.6%) | 26998/525798  (5.1%) | 2.59 [2.36, 2.85] | 42% | 0.01 |
| Using fixed effect meta-analysis model | 16 | 540000 | 1800/14202  (12.6%) | 26998/525798  (5.1%) | 2.61 [2.49, 2.74] | 42% | n/a |
| **NICU ADMISSION** | | | | | | | |
| Excluding studies with serious or critical risk of bias | 17 | 187671 | 511/3134  (16.3%) | 19045/184537  (10.3%) | 1.66 [0.97, 2.83] | 92% | 0.96 |
| Using fixed effect meta-analysis model | 17 | 187671 | 511/3134  (16.3%) | 19045/184537  (10.3%) | 1.37 [1.22, 1.53] | 93% | n/a |
| **1 MIN APGAR** | | | | | | | |
| Excluding studies with serious or critical risk of bias | 9 | 341555 | 485/7032 (6.9%) | 10733/334523 (3.2%) | 2.32 [1.56, 3.47] | 82% | 0.26 |
| Using fixed effect meta-analysis model | 9 | 341555 | 485/7032 (6.9%) | 10733/334523 (3.2%) | 1.44 [1.31, 1.58] | 82% | n/a |
| **5 MIN APGAR** | | | | | | | |
| Excluding studies with serious or critical risk of bias | 15 | 438442 | 185/9316  (2.0%) | 5740/429126  (1.3%) | 3.20 [1.16, 8.85] | 95% | 3.29 |
| Using fixed effect meta-analysis model | 15 | 438442 | 185/9316  (2.0%) | 5740/429126  (1.3%) | 1.05 [0.89, 1.24] | 96% | n/a |

**Table S7.** Subgroup analysis for the primary outcomes.

| **Outcome** | **Subgroup** | **No of studies** | **Total number of participants** | **Idiopathic Polyhydramnios Group Events per total**  **n/N (%)** | **Non- Idiopathic Polyhydramnios Group**  **Events per total**  **n/N (%)** | **RR (95% CI)**  **[Random effect model –**  **Inverse variance method]** | **I^2^** | **τ^2^** |
| --- | --- | --- | --- | --- | --- | --- | --- | --- |
| **Preterm birth** | | | | | | | | |
| Study design | Retrospective | 12 | 290826 | 835/7895  (10.6%) | 16644/282931  (5.9%) | 2.43 [1.52, 3.89] | 94% | 0.55 |
|  | Prospective | 2 | 1012 | 14/248  (5.6%) | 51/764  (6.7%) | 0.79 [0.44, 1.42] | 0% | 0.00 |
| Region | Europe | 5 | 25730 | 101/720  (14.0%) | 2274/25010  (9.1%) | 2.18 [1.56, 3.05] | 24% | 0.04 |
|  | Asia | 6 | 254560 | 693/6827  (10.2%) | 12949/247733  (5.2%) | 3.30 [1.32, 8.24] | 97% | 1.15 |
|  | Americas | 3 | 11548 | 55/596  (9.2%) | 1472/10952  (13.4%) | 0.83 [0.64, 1.08] | 0% | 0.00 |
| Maternal Age | Above 30 | 1 | 31376 | 6/215  (2.8%) | 1262/31161  (4.0%) | 0.69 [0.31, 1.52] | n/a | n/a |
|  | Below or equal to 30 | 6 | 204347 | 717/6912  (10.4%) | 10221/197435  (5.2%) | 3.55 [1.49, 8.43] | 97% | 1.04 |
|  | Unknown | 7 | 56115 | 126/1016  (12.4%) | 5212/55099  (9.5%) | 1.50 [0.95, 2.37] | 75% | 0.23 |
| Year of study | After 2010 | 12 | 290826 | 835/7895  (10.6%) | 16644/282931  (5.9%) | 2.43 [1.52, 3.89] | 94% | 0.55 |
|  | 2010 and before | 2 | 1012 | 14/248  (5.6%) | 51/764  (6.7%) | 0.79 [0.44, 1.42] | 0% | 0.00 |
| Birthweight | More than 3.5kg | 0 | 0 | n/a | n/a | n/a | n/a | n/a |
|  | Between 3kg-3.5kg | 6 | 234723 | 453/6627  (6.8%) | 11455/228096  (5.0%) | 1.99 [1.14, 3.48] | 89% | 0.35 |
|  | Less than or equal to 3kg | 1 | 1000 | 270/500  (54.0%) | 28/500  (5.6%) | 9.64 [6.67, 13.94] | n/a | n/a |
|  | Unknown | 7 | 56115 | 126/1016  (12.4%) | 5212/55099  (9.5%) | 1.50 [0.95, 2.37] | 75% | 0.23 |
| Diagnosis | More than 26 weeks | 5 | 87418 | 363/1399  (25.9%) | 6441/86019  (7.5%) | 1.66 [0.66, 4.14] | 96% | 0.99 |
|  | Less than 26 weeks | 5 | 11667 | 81/717  (11.3%) | 1485/10950  (13.6%) | 1.27 [0.79, 2.06] | 58% | 0.16 |
|  | Unknown | 4 | 192753 | 405/6027  (6.7%) | 8769/186726  (4.7%) | 6.03 [1.30, 27.92] | 93% | 2.19 |
| Definition | AFI | 10 | 99338 | 500/2278  (21.9%) | 7900/97060  (8.1%) | 2.08 [1.11, 3.92] | 94% | 0.91 |
|  | VP | 0 | 0 | n/a | n/a | n/a | n/a | n/a |
|  | Both | 4 | 192500 | 349/5865  (6.0%) | 8795/186635  (4.7%) | 2.10 [0.98, 4.52] | 77% | 0.42 |
| **PIH/PET** | | | | | | | | |
| Study design | Retrospective | 5 | 73831 | 66/1637  (4.0%) | 1623/72194  (2.2%) | 0.93 [0.52, 1.66] | 63% | 0.24 |
|  | Prospective | 4 | 61207 | 22/1446  (1.5%) | 491/59761  (0.8%) | 1.02 [0.64, 1.63] | 0% | 0.00 |
| Region | Europe | 0 | 0 | n/a | n/a | n/a | n/a | n/a |
|  | Asia | 6 | 123422 | 62/2403  (2.6%) | 1459/121019  (1.2%) | 1.24 [0.89, 1.73] | 0% | 0.00 |
|  | Americas | 3 | 11616 | 26/680  (3.8%) | 655/10936  (6.0%) | 0.64 [0.24, 1.73] | 62% | 0.47 |
| Maternal Age | Above 30 | 1 | 31376 | 3/215  (1.4%) | 452/31161  (1.5%) | 0.96 [0.31, 2.97] | n/a | n/a |
|  | Below or equal to 30 | 7 | 73688 | 81/2576  (3.1%) | 1160/71112  (1.6%) | 1.01 [0.66, 1.54] | 46% | 0.13 |
|  | Unknown | 1 | 29974 | 4/292  (1.4%) | 502/29682  (1.7%) | 0.81 [0.30, 2.15] | n/a | n/a |
| Year of study | After 2010 | 5 | 73831 | 66/1637  (4.0%) | 1623/72194  (2.2%) | 0.93 [0.52, 1.66] | 63% | 0.24 |
|  | 2010 and before | 4 | 61207 | 22/1446  (1.5%) | 491/59761  (0.8%) | 1.02 [0.64, 1.63] | 0% | 0.00 |
| Birthweight | More than 3.5kg | 0 | 0 | *n/a* | n/a | n/a | n/a | n/a |
|  | Between 3kg-3.5kg | 5 | 103694 | 40/2106  (1.9%) | 1583/101588  (1.6%) | 0.89 [0.58, 1.38] | 31% | 0.07 |
|  | Less than or equal to 3kg | 2 | 1144 | 38/572  (6.6%) | 22/572  (3.8%) | 1.59 [0.74, 3.41] | 19% | 0.10 |
|  | Unknown | 2 | 30200 | 10/405  (2.5%) | 509/29795  (1.7%) | 0.83 [0.41, 1.70] | 0% | 0.00 |
| Diagnosis | More than 26 weeks | 4 | 123052 | 54/2218  (2.4%) | 1449/120834  (1.2%) | 1.31 [0.91, 1.90] | 7% | 0.01 |
|  | Less than 26 weeks | 3 | 11616 | 26/680  (3.8%) | 655/10936  (6.0%) | 0.64 [0.24, 1.73] | 62% | 0.47 |
|  | Unknown | 2 | 370 | 8/185  (4.3%) | 10/185  (5.4%) | 0.80 [0.32, 1.99] | 0% | 0.00 |
| Definition | AFI | 6 | 73256 | 72/1540  (4.7%) | 1606/71716  (2.2%) | 1.14 [0.85, 1.52] | 0% | 0.00 |
|  | VP | 1 | 135 | 3/50  (6.0%) | 5/85  (5.9%) | 1.02 [0.25, 4.09] | n/a | n/a |
|  | Both | 2 | 61647 | 13/1493  (0.9%) | 503/60154  (0.8%) | 0.50 [0.08, 3.09] | 82% | 1.45 |
| **Labour induction** | | | | | | | | |
| Study design | Retrospective | 7 | 255101 | 1946/6796  (28.6%) | 42383/248305 (17.1%) | 1.50 [1.20, 1.88] | 73% | 0.05 |
|  | Prospective | 1 | 60702 | 277/1211  (22.9%) | 4999/59491  (8.4%) | 2.72 [2.45, 3.03] | n/a | n/a |
| Region | Europe | 2 | 825 | 94/301  (31.2%) | 103/524  (19.7%) | 1.75 [0.81, 3.81] | 90% | 0.28 |
|  | Asia | 5 | 314033 | 2059/7424  (27.7%) | 47132/306609 (15.4%) | 1.91 [1.22, 2.98] | 97% | 0.19 |
|  | Americas | 1 | 945 | 70/282  (24.8%) | 147/663  (22.2%) | 1.12 [0.87, 1.43] | n/a | n/a |
| Maternal Age | Above 30 | 2 | 31658 | 59/309  (19.1%) | 1530/31349  (4.9%) | 2.11 [1.34, 3.34] | 57% | 0.06 |
|  | Below or equal to 30 | 4 | 254069 | 2138/7383  (29.0%) | 44645/246685 (18.1%) | 1.53 [1.02,2.31] | 98% | 0.16 |
|  | Unknown | 2 | 30076 | 26/314  (8.3%) | 1207/29762  (4.1%) | 7.80 [0.16, 383.29] | 87% | 6.97 |
| Year of study | After 2010 | 7 | 255101 | 1946/6796  (28.6%) | 42383/248305 (17.1%) | 1.50 [1.20, 1.88] | 73% | 0.05 |
|  | 2010 and before | 1 | 60702 | 277/1211  (22.9%) | 4999/59491  (8.4%) | 2.72 [2.45, 3.03] | n/a | n/a |
| Birthweight | More than 3.5kg | 1 | 282 | 42/94  (44.7%*)* | 32/188  (17.0%) | 2.63 [1.78, 3.87] | n/a | n/a |
|  | Between 3kg – 3.5kg | 4 | 93566 | 416/1915  (21.7%) | 6715/91651  (7.3%) | 1.57 [0.91, 2.73] | 95% | 0.29 |
|  | Less than or equal to 3kg | 0 | 0 | n/a | n/a | n/a | n/a | n/a |
|  | Unknown | 3 | 221955 | 1765/5998  (29.4%) | 40635/215957 (18.8%) | 1.65 [0.91, 3.00] | 74% | 0.17 |
| Diagnosis | More than 26 weeks | 3 | 122052 | 310/1718  (18.0%) | 7704/120334  (6.4%) | 1.90 [1.17, 3.09] | 83% | 0.15 |
|  | Less than 26 weeks | 2 | 1227 | 112/376  (29.9%) | 179/851  (21.0%) | 1.69 [0.73, 3.90] | 92% | 0.34 |
|  | Unknown | 3 | 192524 | 1801/5913  (30.5%) | 39499/186611  (21.2%) | 1.46 [0.94, 2.26] | 78% | 0.09 |
| Definition | AFI | 4 | 253772 | 1824/6398  (28.5%) | 42204/247374 (17.1%) | 1.44 [1.39, 1.50] | 0% | 0.00 |
|  | VP | 0 | 0 | n/a | n/a | n/a | n/a | n/a |
|  | Both | 4 | 62031 | 399/1609  (24.8%) | 5178/60422  (8.6%) | 2.34 [1.25, 4.37] | 94% | 0.30 |
| **Caesarean section** | | | | | | | | |
| Study design | Retrospective | 15 | 378897 | 2348/8576  (27.3%) | 84904/370321  (23.0%) | 1.64 [1.36, 1.97] | 94% | 0.09 |
|  | Prospective | 10 | 62562 | 546/1884  (29.0%) | 5359/60678  (8.8%) | 1.55 [1.11, 2.15] | 97% | 0.24 |
| Region | Europe | 8 | 26264 | 377/963  (39.1%) | 9467/25301  (37.4%) | 1.43 [0.87, 2.37] | 93% | 0.43 |
|  | Asia | 12 | 402567 | 2169/8569  (25.3%) | 77336/393998 (19.6%) | 1.72 [1.36, 2.16] | 98% | 0.14 |
|  | Americas | 5 | 12628 | 348/928  (37.5%) | 3460/11700  (29.6%) | 1.56 [1.30, 1.89] | 57% | 0.02 |
| Maternal Age (mean) | Above 30 | 4 | 31728 | 86/349  (24.6%) | 1626/31379  (5.2%) | 2.28 [0.94, 5.55] | 94% | 0.69 |
|  | Below or equal to 30 | 15 | 353718 | 2540/9117  (27.8%) | 73962/344601 (21.5%) | 1.65 [1.37, 1.99] | 96% | 0.11 |
|  | Unknown | 6 | 56013 | 268/994  (27.0%) | 14675/55019  (26.7%) | 1.29 [0.79, 2.10] | 92% | 0.31 |
| Year of study | After 2010 | 18 | 379189 | 2442/8745  (27.9%) | 84954/370444 (23.0%) | 1.56 [1.30, 1.87] | 95% | 0.11 |
|  | 2010 and before | 7 | 62270 | 452/1715  (26.4%) | 5309/60555  (8.8%) | 1.76 [1.09, 2.82] | 97% | 0.37 |
| Birthweight | More than 3.5kg | 2 | 361 | 40/123  (32.5%) | 18/238  (7.6%) | 2.81 [0.29, 27.23] | 93% | 2.49 |
|  | Between 3kg – 3.5kg | 12 | 191785 | 1131/2953  (38.3%) | 55101/188832 (29.1%) | 1.74 [1.39, 2.19] | 95% | 0.12 |
|  | Less than or equal to 3kg | 2 | 1144 | 158/572  (27.6%) | 124/572  (21.7%) | 1.28 [1.04, 1.57] | 0% | 0 |
|  | Unknown | 9 | 248169 | 1565/6812  (23.0%) | 35020/241357 (14.5%) | 1.40 [0.99, 1.96] | 97% | 0.23 |
| Diagnosis | More than 26 weeks | 7 | 148199 | 603/2639  (22.8%) | 21355/145560 (14.7%) | 1.35 [0.83, 2.20] | 97% | 0.39 |
|  | Less than 26 weeks | 10 | 205081 | 1734/6947  (25.0%) | 23774/198134 (12.0%) | 1.79 [1.48, 2.18] | 83% | 0.05 |
|  | Unknown | 8 | 88179 | 557/874  (63.7%) | 45134/87305  (51.7%) | 1.56 [1.23, 1.98] | 95% | 0.08 |
| Definition | AFI | 17 | 186958 | 1157/2960  (39.1%) | 64592/183998 (35.1%) | 1.49 [1.26, 1.78] | 92% | 0.10 |
|  | VP | 1 | 135 | 12/50  (24%) | 11/85  (13.0%) | 1.85 [0.88, 3.89] | n/a | n/a |
|  | Both | 7 | 254366 | 1725/7450  (23.6%) | 25660/246916 (10.4%) | 1.88 [1.32, 2.68] | 98% | 0.18 |
| **Placental abruption** | | | | | | | | |
| Study design | Retrospective | 6 | 97623 | 52/1764  (2.9%) | 379/95859  (0.4%) | 2.95 [1.62, 5.38] | 29% | 0.16 |
|  | Prospective | 3 | 61396 | 12/1358  (0.9%) | 161/60038  (0.3%) | 3.41 [1.87, 6.19] | 0% | 0.00 |
| Region | Europe | 1 | 24509 | 6/295  (2.0%) | 168/24214  (0.7%) | 2.93 [1.31, 6.56] | n/a | n/a |
|  | Asia | 5 | 123280 | 55/2332  (2.4%) | 284/120948  (0.2%) | 3.80 [2.46, 5.85] | 0% | 0.00 |
|  | Americas | 3 | 11230 | 3/495  (0.6%) | 88/10735  (0.8%) | 1.09 [0.20, 5.96] | 24% | 0.49 |
| Maternal Age | Above 30 | 1 | 31376 | 2/215  (0.9%) | 61/31161  (0.2%) | 4.75 [1.17, 19.31] | n/a | n/a |
|  | Below or equal to 30 | 5 | 72601 | 55/2223  (2.5%) | 257/70378  (0.4%) | 3.05 [1.54, 6.04] | 39% | 0.21 |
|  | Unknown | 3 | 55042 | 7/684  (1.0%) | 222/54358  (0.4%) | 2.75 [1.30, 5.80] | 0% | 0.00 |
| Year of study | After 2010 | 6 | 97623 | 52/1764  (2.9%) | 379/95859  (0.4%) | 2.95 [1.62, 5.38] | 29% | 0.16 |
|  | 2010 and before | 3 | 61396 | 12/1358  (0.9%) | 161/60038  (0.3%) | 3.41 [1.87, 6.19] | 0% | 0.00 |
| Birthweight | More than 3.5kg | 0 | 0 | n/a | n/a | n/a | n/a | n/a |
|  | Between 3kg-3.5kg | 5 | 102977 | 22/1938  (1.1%) | 310/101039  (0.3%) | 2.88 [1.29, 6.44] | 35% | 0.28 |
|  | Less than or equal to 3kg | 1 | 1000 | 35/500  (7.0%) | 8/500  (1.6%) | 4.38 [2.05, 9.34] | n/a | n/a |
|  | Unknown | 3 | 55042 | 7/684  (1.0%) | 222/54358  (0.4%) | 2.75 [1.30, 5.80] | 0% | 0.00 |
| Diagnosis | More than 26 weeks | 6 | 148120 | 55/2610  (2.1%) | 452/145510  (0.3%) | 3.50 [2.38, 5.14] | 0% | 0.00 |
|  | Less than 26 weeks | 2 | 10671 | 3/398  (0.8%) | 88/10273  (0.9%) | 1.09 [0.20, 5.96] | 24% | 0.49 |
|  | Unknown | 1 | 228 | 6/114  (5.2%) | 0/114  (0%) | 13.00 [0.74, 228.09] | n/a | n/a |
| Definition | AFI | 7 | 98182 | 52/1861  (2.8%) | 379/96321  (0.4%) | 2.95 [1.62, 5.38] | 29% | 0.16 |
|  | VP | 1 | 135 | 1/50  (2.0%) | 0/85  (0%) | 5.06 [0.21, 121.87] | n/a | n/a |
|  | Both | 1 | 60702 | 11/1211  (0.9%) | 161/59491  (0.3%) | 3.36 [1.83, 6.17] | n/a | n/a |
| **Malpresentation** | | | | | | | | |
| Study design | Retrospective | 8 | 143905 | 169/1956  (8.6%) | 5611/141949  (4.0%) | 1.99 [1.31, 3.02] | 69% | 0.20 |
|  | Prospective | 4 | 61849 | 96/1509  (6.4%) | 1772/60340  (2.9%) | 2.29 [1.87, 2.81] | 0% | 0.00 |
| Region | Europe | 4 | 25786 | 59/743  (7.9%) | 2097/25043  (8.4%) | 1.38 [1.07, 1.79] | 0% | 0.00 |
|  | Asia | 5 | 178821 | 192/2424  (7.9%) | 5265/176397  (3.0%) | 2.76 [2.04, 3.75] | 46% | 0.05 |
|  | Americas | 3 | 1147 | 14/298  (4.7%) | 21/849  (2.5%) | 2.18 [1.05, 4.52] | 0% | 0.00 |
| Maternal Age | Above 30 | 0 | 0 | n/a | n/a | n/a | n/a | n/a |
|  | Below or equal to 30 | 8 | 150259 | 210/2630  (8.0%) | 5258/147629  (3.6%) | 2.02 [1.43, 2.85] | 57% | 0.10 |
|  | Unknown | 4 | 55495 | 55/835  (6.6%) | 2125/54660  (3.9%) | 2.40 [1.23, 4.67] | 60% | 0.27 |
| Year of study | After 2010 | 8 | 143905 | 169/1956  (8.6%) | 5611/141949  (4.0%) | 1.99 [1.31, 3.02] | 69% | 0.20 |
|  | 2010 and before | 4 | 61849 | 96/1509  (6.4%) | 1772/60340  (2.9%) | 2.29 [1.87, 2.81] | 0% | 0.00 |
| Birthweight | More than 3.5kg | 0 | 0 | n/a | n/a | n/a | n/a | n/a |
|  | Between 3kg-3.5kg | 7 | 149259 | 136/2130  (6.4%) | 5240/147129  (3.6%) | 1.80 [1.31, 2.47] | 38% | 0.06 |
|  | Less than or equal to 3kg | 1 | 1000 | 74/500  (14.8%) | 18/500  (3.6%) | 4.11 [2.49, 6.78] | n/a | n/a |
|  | Unknown | 4 | 55495 | 55/835  (6.6%) | 2125/54660  (3.9%) | 2.40 [1.23, 4.67] | 60% | 0.27 |
| Diagnosis | More than 26 weeks | 6 | 117318 | 212/2577  (8.2%) | 3909/114741  (3.4%) | 2.18 [1.48, 3.23] | 74% | 0.14 |
|  | Less than 26 weeks | 3 | 748 | 11/260  (4.2%) | 8/488  (1.6%) | 2.57 [1.02, 6.48] | 0% | 0.00 |
|  | Unknown | 3 | 87688 | 42/628  (6.7%) | 3466/87060  (4.0%) | 1.68 [0.82, 3.42] | 59% | 0.23 |
| Definition | AFI | 10 | 144917 | 182/2204  (8.3%) | 5631/142713  (3.9%) | 2.03 [1.40, 2.94] | 63% | 0.18 |
|  | VP | 1 | 135 | 1/50  (2.0%) | 1/85  (1.2%) | 1.70 [0.11, 26.58] | n/a | n/a |
|  | Both | 1 | 60702 | 82/1211  (6.8%) | 1751/59491  (2.9%) | 2.30 [1.86, 2.85] | n/a | n/a |
| **PPH** | | | | | | | | |
| Study design | Retrospective | 7 | 160976 | 123/2058  (6.0%) | 4115/158918  (2.6%) | 2.55 [1.34, 4.85] | 84% | 0.57 |
|  | Prospective | 2 | 684 | 2/147  (1.4%) | 3/537  (0.6%) | 2.03 [0.33, 12.36] | 0% | 0.00 |
| Region | Europe | 0 | 0 | n/a | n/a | n/a | n/a | n/a |
|  | Asia | 5 | 149495 | 64/1428  (4.5%) | 4033/148067  (2.7%) | 1.72 [1.11, 2.68] | 37% | 0.09 |
|  | Americas | 4 | 12165 | 61/777  (7.9%) | 85/11388  (0.7%) | 3.74 [0.89, 15.68] | 88% | 1.52 |
| Maternal Age | Above 30 | 1 | 31376 | 6/215  (2.8%) | 688/31161  (2.2%) | 1.26 [0.57, 2.79] | n/a | n/a |
|  | Below or equal to 30 | 6 | 99761 | 111/1601  (6.9%) | 2871/98160  (2.9%) | 3.32 [1.49, 7.37] | 85% | 0.67 |
|  | Unknown | 2 | 30523 | 8/389  (2.1%) | 559/30134  (1.9%) | 1.35 [0.67, 2.72] | 0% | 0.00 |
| Year of study | After 2010 | 7 | 160976 | 123/2058  (6.0%) | 4115/158918  (2.6%) | 2.55 [1.34, 4.85] | 84% | 0.57 |
|  | 2010 and before | 2 | 684 | 2/147  (1.4%) | 3/537  (0.6%) | 2.03 [0.33, 12.36] | 0% | 0.00 |
| Birthweight | More than 3.5kg | 0 | 0 | *n/a* | n/a | n/a | n/a | n/a |
|  | Between 3kg-3.5kg | 6 | 130137 | 84/1316  (6.4%) | 3549/128821  (2.8%) | 2.75 [1.19, 6.36] | 86% | 0.75 |
|  | Less than or equal to 3kg | 1 | 1000 | 33/500  (6.6%) | 10/500  (2.0%) | 3.30 [1.64, 6.62] | n/a | n/a |
|  | Unknown | 2 | 30523 | 8/389  (2.1%) | 559/30134  (1.9%) | 1.35 [0.67, 2.72] | 0% | 0.00 |
| Diagnosis | More than 26 weeks | 4 | 62899 | 47/1104  (4.3%) | 1257/61795  (2.0%) | 1.81 [1.05, 3.13] | 34% | 0.10 |
|  | Less than 26 weeks | 3 | 11616 | 60/680  (8.8%) | 83/10936  (0.8%) | 4.11 [0.78, 21.66] | 92% | 1.71 |
|  | Unknown | 2 | 87145 | 18/421  (4.3%) | 2778/86724  (3.2%) | 2.01 [0.49, 8.20] | 33% | 0.57 |
| Definition | AFI | 7 | 160580 | 77/1873  (4.1%) | 4060/158707  (2.6%) | 2.72 [1.20, 6.13] | 84% | 0.87 |
|  | VP | 1 | 135 | 1/50  (2.0%) | 1/85  (1.2%) | 1.70 [0.11, 26.58] | n/a | n/a |
|  | Both | 1 | 945 | 47/282  (16.7%) | 57/663  (8.6%) | 1.94 [1.35, 2.78] | n/a | n/a |
| **Perinatal mortality** | | | | | | | | |
| Study design | Retrospective | 11 | 185416 | 139/2388  (5.8%) | 346/183028  (0.2%) | 4.60 [1.86, 11.40] | 48% | 1.00 |
|  | Prospective | 4 | 61849 | 43/1509  (2.8%) | 335/60340  (0.6%) | 6.03 [4.39, 8.29] | 0% | 0.00 |
| Region | Europe | 4 | 25187 | 4/513  (0.8%) | 123/24674  (0.5%) | 2.28 [0.52, 9.97] | 0% | 0.00 |
|  | Asia | 6 | 210197 | 173/2639  (6.6%) | 525/207558  (0.3%) | 7.92 [3.73, 16.82] | 54% | 0.39 |
|  | Americas | 5 | 11881 | 5/745  (0.7%) | 33/11136  (0.3%) | 2.23 [0.52, 9.57] | 0% | 0.00 |
| Maternal Age | Above 30 | 1 | 31376 | 1/215  (0.5%) | 42/31161  (0.1%) | 3.45 [0.48, 24.96] | n/a | n/a |
|  | Below or equal to 30 | 7 | 159678 | 177/2589  (6.8%) | 461/157089  (0.3%) | 7.91 [3.73, 16.77] | 52% | 0.41 |
|  | Unknown | 7 | 56211 | 4/1093  (0.4%) | 178/55118  (0.3%) | 1.69 [0.46, 6.22] | 0% | 0.00 |
| Year of study | After 2010 | 10 | 185218 | 137/2289  (6.0%) | 346/182929  (0.2%) | 4.50 [1.70, 11.92] | 53% | 1.16 |
|  | 2010 and before | 5 | 62047 | 45/1608  (2.8%) | 335/60439  (0.6%) | 6.02 [4.39, 8.26] | 0% | 0.00 |
| Birthweight | More than 3.5kg | 1 | 198 | 2/99  (2.0%) | 0/99  (0/0%) | 5.00 [0.24, 102.83] | n/a | n/a |
|  | Between 3kg-3.5kg | 7 | 190054 | 80/2304  (3.5%) | 498/187750  (0.3%) | 5.73 [3.45, 9.49] | 10% | 0.07 |
|  | Less than or equal to 3kg | 1 | 1000 | 98/500  (19.6%) | 5/500  (1.0%) | 19.60 [8.05, 47.73] | n/a | n/a |
|  | Unknown | 6 | 56013 | 2/994  (0.2%) | 178/55019  (0.3%) | 1.32 [0.31, 5.59] | 0% | 0.00 |
| Diagnosis | More than 26 weeks | 6 | 148120 | 140/2610  (5.4%) | 558/145510  (0.4%) | 4.84 [1.75, 13.44] | 69% | 0.73 |
|  | Less than 26 weeks | 6 | 11802 | 7/767  (0.9%) | 35/11035  (0.3%) | 2.84 [0.86, 9.45] | 0% | 0.00 |
|  | Unknown | 3 | 87343 | 35/520  (6.7%) | 88/86823  (0.1%) | 9.67 [2.66, 35.18] | 11% | 0.18 |
| Definition | AFI | 11 | 185910 | 137/2477  (5.5%) | 344/183433  (0.2%) | 4.62 [1.63, 13.12] | 58% | 1.30 |
|  | VP | 1 | 135 | 2/50  (4.0%) | 0/85  (0.0%) | 8.43 [0.41, 172.17] | n/a | n/a |
|  | Both | 3 | 61220 | 43/1370  (3.1%) | 337/59850  (0.6%) | 5.95 [4.34, 8.16] | 0% | 0.00 |
| **Stillbirth** | | | | | | | | |
| Study design | Retrospective | 8 | 73830 | 65/1727  (3.8%) | 113/72103  (0.2%) | 5.80 [2.38, 14.14] | 15% | 0.25 |
|  | Prospective | 4 | 61849 | 9/1509  (0.6%) | 108/60340  (0.2%) | 3.38 [1.61, 7.07] | 0% | 0.00 |
| Region | Europe | 2 | 518 | 2/159  (1.3%) | 1/359  (0.3%) | 4.75 [0.53, 42.32] | 0% | 0.00 |
|  | Asia | 5 | 123280 | 67/2332  (2.9%) | 201/120948  (0.2%) | 5.71 [1.87, 17.44] | 54% | 0.79 |
|  | Americas | 5 | 11881 | 5/745  (0.7%) | 19/11136  (0.2%) | 3.03 [0.70, 13.07] | 0% | 0.00 |
| Maternal Age | Above 30 | 1 | 31376 | 1/215  (0.5%) | 39/31161  (0.1%) | 3.72 [0.51, 26.93] | n/a | n/a |
|  | Below or equal to 30 | 5 | 72601 | 69/2223  (3.1%) | 129/70378  (0.2%) | 6.28 [2.02, 19.52] | 54% | 0.81 |
|  | Unknown | 6 | 31702 | 4/798  (0.5%) | 53/30904  (0.2%) | 3.04 [0.68, 13.54] | 0% | 0.00 |
| Year of study | After 2010 | 7 | 73632 | 63/1628  (3.9%) | 113/72004  (0.2%) | 5.57 [2.03, 15.33] | 27% | 0.49 |
|  | 2010 and before | 5 | 62047 | 11/1608  (0.7%) | 108/60439  (0.2%) | 3.45 [1.68, 7.08] | 0% | 0.00 |
| Birthweight | More than 3.5kg | 1 | 198 | 2/99  (2.0%) | 0/99  (0.0%) | 5.00 [0.24,102.83] | n/a | n/a |
|  | Between 3kg-3.5kg | 5 | 102977 | 18/1938  (0.9%) | 166/101039  (0.2%) | 3.39 [1.79, 6.42] | 0% | 0.00 |
|  | Less than or equal to 3kg | 1 | 1000 | 52/500  (10.4%) | 2/500  (0.4%) | 26.00 [6.37, 106.16] | n/a | n/a |
|  | Unknown | 5 | 31504 | 2/699  (0.3%) | 53/30805  (0.2%) | 2.59 [0.46, 14.43] | 0% | 0.00 |
| Diagnosis | More than 26 weeks | 5 | 123611 | 60/2315  (2.6%) | 201/121296  (0.2%) | 5.02 [1.42, 17.70] | 62% | 0.96 |
|  | Less than 26 weeks | 5 | 11642 | 5/708  (0.7%) | 20/10934  (0.2%) | 3.25 [0.86, 12.25] | 0% | 0.00 |
|  | Unknown | 2 | 426 | 9/213  (4.2%) | 0/213  (0%) | 8.94 [1.12, 71.19] | 0% | 0.00 |
| Definition | AFI | 8 | 74324 | 63/1816  (3.5%) | 112/72508  (0.2%) | 5.46 [1.73, 17.29] | 39% | 0.78 |
|  | VP | 1 | 135 | 2/50  (4.0%) | 0/85  (0%) | 8.43 [0.41, 172.17] | n/a | n/a |
|  | Both | 3 | 61220 | 9/1370  (0.7%) | 109/59850  (0.2%) | 3.33 [1.62, 6.83] | 0% | 0.00 |
| **Macrosomia** | | | | | | | | |
| Study design | Retrospective | 13 | 538961 | 1750/13938  (12.6%) | 26940/525023  (5.1%) | 2.59 [2.34, 2.87] | 51% | 0.01 |
|  | Prospective | 3 | 1039 | 50/264  (18.9%) | 58/778  (7.4%) | 2.61 [1.80, 3.79] | 0% | 0.00 |
| Region | Europe | 4 | 25500 | 127/705  (18.0%) | 2625/24795  (10.6%) | 2.13 [1.48, 3.08] | 44% | 0.06 |
|  | Asia | 7 | 501980 | 1506/12603  (11.9%) | 23217/489377  (4.7%) | 2.67 [2.38, 3.01] | 54% | 0.01 |
|  | Americas | 5 | 12520 | 167/894  (18.7%) | 1156/11626  (9.9%) | 2.73 [2.12, 3.53] | 27% | 0.02 |
| Maternal Age | Above 30 | 0 | 0 | n/a | n/a | n/a | n/a | n/a |
|  | Below or equal to 30 | 9 | 484088 | 1626/13185  (12.3%) | 24055/470903  (5.1%) | 2.61 [2.49, 2.74] | 0% | 0.00 |
|  | Unknown | 7 | 55912 | 174/1017  (17.1%) | 2943/54895  (5.4%) | 2.83 [1.90, 4.21] | 70% | 0.16 |
| Year of study | After 2010 | 13 | 538961 | 1750/13938  (12.6%) | 26940/525023  (5.1%) | 2.59 [2.34, 2.87] | 51% | 0.01 |
|  | 2010 and before | 3 | 1039 | 50/264  (18.9%) | 58/775  (7.5%) | 2.61 [1.80, 3.79] | 0% | 0.00 |
| Birthweight | More than 3.5kg | 0 | 0 | n/a | n/a | n/a | n/a | n/a |
|  | Between 3kg-3.5kg | 8 | 291236 | 872/7017  (12.4%) | 15457/284219  (5.4%) | 2.63 [2.46, 2.81] | 0% | 0.00 |
|  | Less than or equal to 3kg | 1 | 1000 | 75/500  (15.0%) | 33/500  (6.6%) | 2.27 [1.54, 3.36] | n/a | n/a |
|  | Unknown | 7 | 247764 | 853/6685  (12.8%) | 11508/241079  (4.8%) | 2.66 [2.10, 3.37] | 69% | 0.05 |
| Diagnosis | More than 26 weeks | 4 | 56042 | 184/1184  (15.5%) | 2938/54858  (5.4%) | 2.70 [1.97, 3.69] | 63% | 0.06 |
|  | Less than 26 weeks | 6 | 12409 | 179/1000  (17.9%) | 1140/11409  (10.0%) | 2.52 [1.75, 3.63] | 54% | 0.09 |
|  | Unknown | 6 | 471549 | 1437/12018  (12.0%) | 22920/459531  (5.0%) | 2.63 [2.44, 2.83] | 23% | 0.00 |
| Definition | AFI | 12 | 346785 | 1072/8070  (13.3%) | 18723/338715  (5.5%) | 2.58 [2.43, 2.74] | 0% | 0.00 |
|  | VP | 0 | 0 | n/a | n/a | n/a | n/a | n/a |
|  | Both | 4 | 193215 | 728/6132  (11.9%) | 8275/187083  (4.4%) | 3.05 [1.64, 5.68] | 80% | 0.27 |
| **NICU admission** | | | | | | | | |
| Study design | Retrospective | 13 | 186988 | 498/2872  (17.3%) | 19027/184116 (10.3%) | 1.77 [0.97, 3.21] | 94% | 1.00 |
|  | Prospective | 4 | 683 | 13/262  (5.0%) | 18/421  (4.3%) | 1.14 [0.56, 2.35] | 0% | 0.00 |
| Region | Europe | 7 | 26071 | 81/837  (9.7%) | 1456/25234  (5.8%) | 1.90 [1.03, 3.50] | 65% | 0.38 |
|  | Asia | 6 | 149639 | 297/1500  (19.8%) | 16154/148139 (10.9%) | 1.98 [0.44, 8.91] | 97% | 3.15 |
|  | Americas | 4 | 11961 | 133/797  (16.7%) | 1435/11164  (12.9%) | 1.09 [0.53, 2.22] | 87% | 0.36 |
| Maternal Age | Above 30 | 2 | 31658 | 32/309  (10.4%) | 14465/31349  (46.1%) | 0.46 [0.04, 4.66] | 97% | 2.73 |
|  | Below or equal to 30 | 9 | 100532 | 415/1912  (21.%) | 1725/98620  (1.7%) | 2.93 [1.53, 5.63] | 90% | 0.68 |
|  | Unknown | 6 | 55481 | 64/913  (7.0%) | 2855/54568  (5.2%) | 1.23 [0.74, 2.04] | 53% | 0.18 |
| Year of study | After 2010 | 14 | 187047 | 502/2895  (17.3%) | 19030/184152 (10.3%) | 1.78 [1.00, 3.17] | 94% | 0.99 |
|  | 2010 and before | 3 | 624 | 9/239  (3.8%) | 15/385  (3.9%) | 0.92 [0.40, 2.13] | 0% | 0.00 |
| Birthweight | More than 3.5kg | 1 | 282 | 18/94  (19.1%) | 24/188  (12.8%) | 1.50 [0.86, 2.62] | n/a | n/a |
|  | Between 3kg – 3.5kg | 9 | 130791 | 210/1571  (13.4%) | 16135/129220 (12.5%) | 1.52 [0.65, 3.54] | 94% | 1.32 |
|  | Less than or equal to 3kg | 2 | 1144 | 220/572  (38.5%) | 33/572  (5.8%) | 6.58 [4.67, 9.27] | 0% | 0.00 |
|  | Unknown | 5 | 55454 | 63/897  (7.0%) | 2853/54557  (5.2%) | 1.30 [0.77, 2.19] | 58% | 0.18 |
| Diagnosis | More than 26 weeks | 4 | 86859 | 263/1302  (20.2%) | 17297/85557  (20.2%) | 0.99 [0.19, 5.12] | 98% | 2.77 |
|  | Less than 26 weeks | 8 | 12921 | 182/1109  (16.4%) | 1482/11812  (12.5%) | 1.42 [0.91, 2.22] | 74% | 0.22 |
|  | Unknown | 5 | 87891 | 66/723  (9.1%) | 266/87168  (0.3%) | 4.86 [2.20, 10.71] | 31% | 0.25 |
| Definition | AFI | 13 | 185926 | 432/2599  (16.6%) | 18903/183327 (10.3%) | 1.71 [0.86, 3.39] | 94% | 1.23 |
|  | VP | 0 | 0 | n/a | n/a | n/a | n/a | n/a |
|  | Both | 4 | 1745 | 79/535  (14.8%) | 142/1210  (11.7%) | 1.51 [0.75, 3.03] | 75% | 0.34 |
| **5m APGAR** | | | | | | | | |
| Study design | Retrospective | 10 | 376411 | 126/7665  (1.6%) | 5664/368746  (1.5%) | 2.17 [0.93, 5.04] | 89% | 1.23 |
|  | Prospective | 5 | 62031 | 59/1651  (3.6%) | 76/60380  (0.1%) | 5.08 [0.89, 29.11] | 89% | 3.23 |
| Region | Europe | 5 | 25673 | 36/825  (4.4%) | 1072/24848  (4.3%) | 2.78 [0.69, 11.29] | 66% | 1.47 |
|  | Asia | 7 | 401221 | 124/7895  (1.6%) | 4395/393326  (1.1%) | 4.23 [0.60, 30.05] | 98% | 6.27 |
|  | Americas | 3 | 11548 | 25/596  (4.2%) | 273/10952  (2.5%) | 1.97 [1.31, 2.97] | 0% | 0.00 |
| Maternal Age | Above 30 | 1 | 31376 | 3/215  (1.4%) | 126/31161  (0.4%) | 3.45 [1.11, 10.76] | n/a | n/a |
|  | Below or equal to 30 | 9 | 351283 | 163/8122  (2.0%) | 4381/343161  (1.3%) | 7.18 [1.58, 32.58] | 97% | 4.43 |
|  | Unknown | 5 | 55783 | 19/979  (1.9%) | 1233/54804  (2.2%) | 0.86 [0.53, 1.38] | 0% | 0.00 |
| Year of study | After 2010 | 11 | 376584 | 138/7785  (1.8%) | 5664/368799  (1.5%) | 2.38 [1.04, 5.42] | 88% | 1.26 |
|  | 2010 and before | 4 | 61858 | 47/1531  (3.1%) | 76/60327  (0.1%) | 4.40 [0.60, 32.22] | 91% | 3.63 |
| Birthweight | More than 3.5kg | 0 | 0 | n/a | n/a | n/a | n/a | n/a |
|  | Between 3kg-3.5kg | 9 | 382515 | 160/8265  (1.9%) | 4506/374250  (1.2%) | 6.65 [1.53, 29.00] | 97% | 4.25 |
|  | Less than or equal to 3kg | 1 | 144 | 6/72  (8.3%) | 1/72  (1.4%) | 6.00 [0.74, 48.59] | n/a | n/a |
|  | Unknown | 5 | 55783 | 19/979  (1.9%) | 1233/54804  (2.2%) | 0.86 [0.53, 1.38] | 0% | 0.00 |
| Diagnosis | More than 26 weeks | 5 | 147120 | 51/2110  (2.4%) | 1410/145010  (1.0%) | 2.25 [0.31, 16.50] | 96% | 4.68 |
|  | Less than 26 weeks | 5 | 11610 | 41/822  (5.0%) | 265/10788  (2.5%) | 2.07 [1.05, 4.08] | 24% | 0.16 |
|  | Unknown | 5 | 279712 | 93/6384  (1.5%) | 4065/273328  (1.5%) | 5.44 [0.81, 36.59] | 92% | 3.79 |
| Definition | AFI | 12 | 185572 | 96/2277  (4.2%) | 1861/183295  (1.0%) | 2.83 [1.51, 5.31] | 64% | 0.59 |
|  | VP | 0 | 0 | n/a | n/a | n/a | n/a | n/a |
|  | Both | 3 | 252870 | 89/7039  (1.3%) | 3879/245831  (1.6%) | 2.17 [0.08, 56.19] | 99% | 8.08 |
| **1m APGAR** |  |  |  |  |  |  |  |  |
| Study design | Retrospective | 7 | 340823 | 433/6815  (6.4%) | 10657/334008 (3.2%) | 2.35 [1.42, 3.88] | 85% | 0.35 |
|  | Prospective | 2 | 732 | 52/217  (24.0%) | 76/515  (14.8%) | 2.59 [0.75, 8.90] | 68% | 0.58 |
| Region | Europe | 3 | 621 | 55/323  (17.0%) | 22/298  (7.4%) | 2.36 [1.27, 4.39] | 28% | 0.09 |
|  | Asia | 5 | 340375 | 405/6612  (6.1%) | 10637/333763  (3.2%) | 2.57 [1.34, 4.94] | 89% | 0.47 |
|  | Americas | 1 | 559 | 25/97  (25.8%) | 74/462  (16.0%) | 1.61 [1.08, 2.39] | n/a | n/a |
| Maternal Age | Above 30 | 1 | 31376 | 10/215  (4.7%) | 647/31161  (2.1%) | 2.24 [1.22, 4.12] | n/a | n/a |
|  | Below or equal to 30 | 5 | 279358 | 430/6284  (6.8%) | 9317/273074  (3.4%) | 3.36 [1.50, 7.50] | 90% | 0.69 |
|  | Unknown | 3 | 30821 | 45/533  (8.4%) | 769/30288  (2.5%) | 1.55 [1.13, 2.13] | 0% | 0.00 |
| Year of study | After 2010 | 8 | 340996 | 460/6935  (6.6%) | 10659/334061  (3.2%) | 2.52 [1.54, 4.13] | 84% | 0.37 |
|  | 2010 and before | 1 | 559 | 25/97  (25.8%) | 74/462  (16.0%) | 1.61 [1.08, 2.39] | n/a | n/a |
| Birthweight | More than 3.5kg | 0 | 0 | n/a | n/a | n/a | n/a | n/a |
|  | Between 3kg-3.5kg | 6 | 310734 | 440/6499  (6.8%) | 9964/304235  (3.3%) | 3.08 [1.60, 5.91] | 89% | 0.53 |
|  | Less than or equal to 3kg | 0 | 0 | n/a | n/a | n/a | n/a | n/a |
|  | Unknown | 3 | 30821 | 45/533  (8.4%) | 769/30288  (2.5%) | 1.55 [1.13, 2.13] | 0% | 0.00 |
| Diagnosis | More than 26 weeks | 3 | 61909 | 45/604  (7.5%) | 1409/61305  (2.3%) | 1.70 [1.27, 2.28] | 0% | 0.00 |
|  | Less than 26 weeks | 3 | 621 | 55/323  (17.0%) | 22/298  (7.4%) | 2.36 [1.27, 4.39] | 28% | 0.09 |
|  | Unknown | 3 | 279025 | 385/6105  (6.3%) | 9302/272920  (3.4%) | 3.40 [1.05, 10.96] | 94% | 0.98 |
| Definition | AFI | 7 | 149387 | 143/1204  (11/9%) | 2190/148183  (1.5%) | 2.78 [1.78, 4.34] | 70% | 0.24 |
|  | VP | 0 | 0 | n/a | n/a | n/a | n/a | n/a |
|  | Both | 2 | 192168 | 342/5828  (5.9%) | 8543/186340  (4.6%) | 1.28 [1.15, 1.42] | 0% | 0.00 |

**SUPPLEMENTARY FIGURES**

**Figure S1.** Association of idiopathic polyhydramnios with the risk of a) Malpresentation b) Transverse lie c) Breech presentation d) Epidural analgesia e) Cephalopelvic disproportion f) Cord prolapse g) Low birth weight h) Macrosomia.

1. Malpresentation


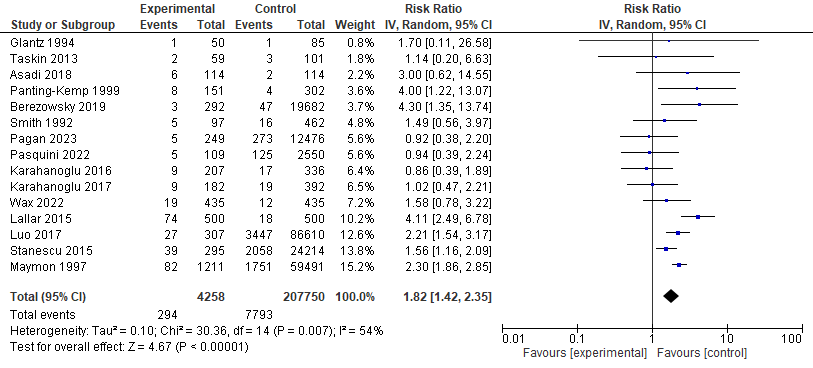


1. Transverse lie


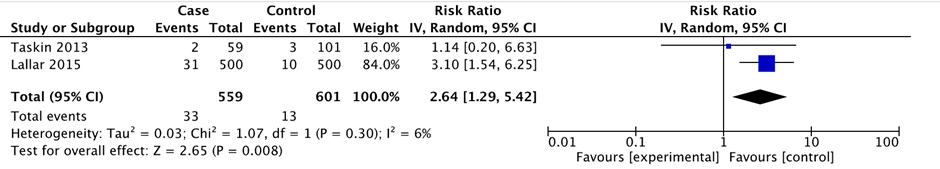


1. Breech


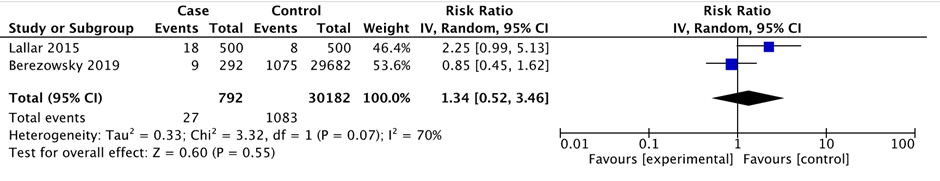


1. Epidural analgesia


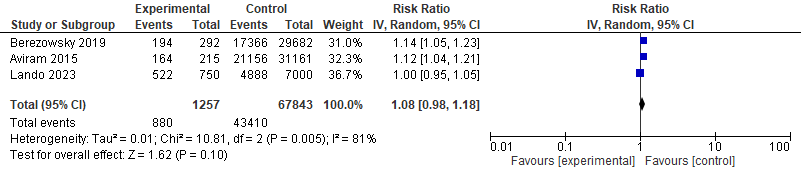


1. Cephalopelvic disproportion


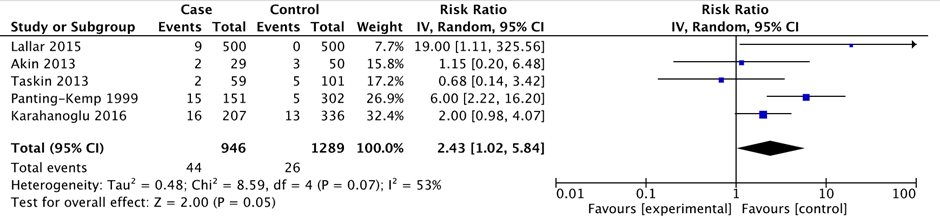


1. Cord prolapse


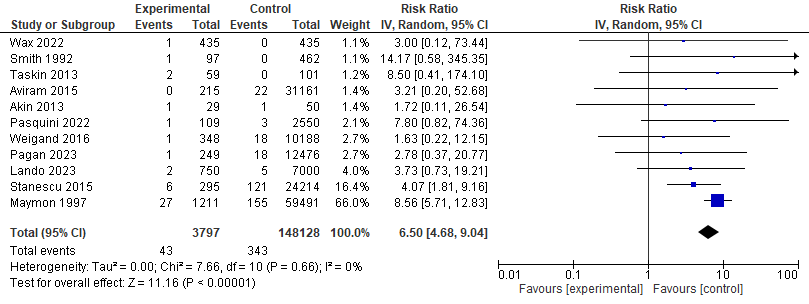


1. Low birth weight


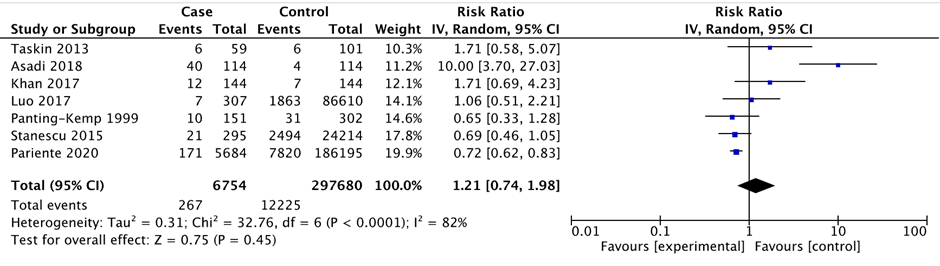


1. Macrosomia


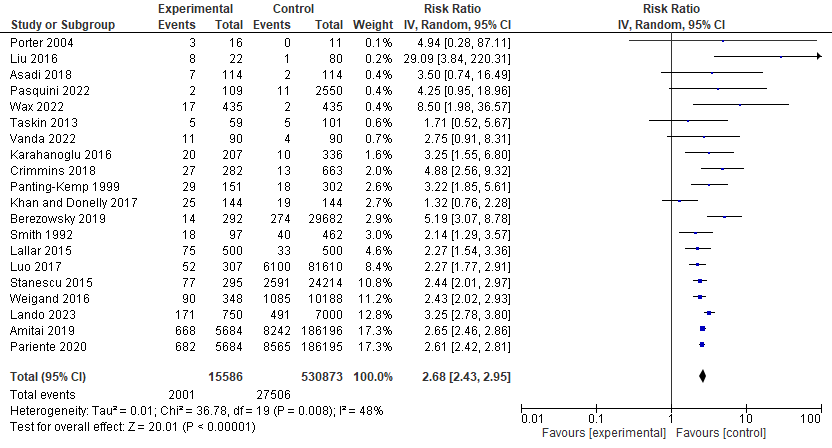


Abbreviations

95% CI: 95% confidence interval

RR: risk ratio

IV: inverse variance
